# Recent Advances in Autophagy and Immunotherapy for the Clearance of Aggregated α-Synuclein in Parkinson’s Disease

**DOI:** 10.14336/AD.2025.0642

**Published:** 2026-08-06

**Authors:** Khoshnur Jannat, Sang-Bong Lee, Hong-Ryeol Jung, Rengasamy Balakrishnan, Yon-Suk Kim, Mujeeb Ur Rahman, Dong-Kug Choi

**Affiliations:** ^1^Department of Applied Life Sciences, Graduate School, Konkuk University, Chungju 27478, South Korea.; ^2^Department of Biotechnology, College of Biomedical and Health Science, Research Institute of Inflammatory Disease (RID), Konkuk University, Chungju 27478, South Korea.

**Keywords:** alpha-synuclein, Parkinson’s disease;, small molecule, peptide, immunotherapy

## Abstract

Parkinson’s disease is a neurodegenerative condition characterized by the accumulation of misfolded and aggregated α-synuclein in Lewy bodies and neurites. These protein aggregates contribute to neurodegeneration and motor symptoms such as bradykinesia, rigidity, and tremor. While the ubiquitin-proteasome system degrades soluble α-synuclein, aggregated and oligomeric forms are primarily cleared via the autophagy-lysosomal pathway. Mutations of the *SNCA* gene exacerbate α-synuclein aggregation and significantly impair its clearance, highlighting the importance of targeting toxic α-synuclein species. Strategies such as promoting autophagosome formation via 5’-AMP-activated protein kinase (AMPK) and mechanistic target of rapamycin complex 1 (mTORC1) or facilitating autophagosome maturation via RAB7-a member of the RAS oncogene family-and related effectors, have shown promise in enhancing autophagy and reducing α-synuclein pathology. Pharmacological agents such as rapamycin, trehalose, and nilotinib have demonstrated preclinical efficacy in enhancing α-synuclein clearance and alleviating disease features. Concurrently, immunotherapy approaches, including passive and active immunization, aim to enhance the immune system’s ability to recognize and eliminate toxic α-synuclein species. Emerging strategies such as peptide-based therapies aim to inhibit aggregation or promote degradation of α-synuclein. At the same time, nanotechnology enables the targeted delivery of therapeutic agents across the blood-brain barrier with improved efficiency. Additionally, novel AUTOTAC (autophagy-targeting chimera) platforms offer a precision strategy to tag α-synuclein for autophagic degradation. This review explores many advances in autophagy-mediated aggregated α-synuclein clearance, emphasizing its potential as a therapeutic strategy to address the limitations of current symptomatic treatments and slow the progression of Parkinson’s disease.

## INTRODUCTION

Parkinson’s disease (PD) is a progressive neurodegenerative disease (NDD) characterized by the accumulation of misfolded and aggregated α-synuclein in Lewy bodies and neurites, which are pathological hallmarks of the disease. α-synuclein aggregation disrupts cellular homeostasis, contributing to neurodegeneration and clinical manifestations such as bradykinesia, rigidity, and tremor [1,2]. Under normal conditions, soluble α-synuclein is primarily degraded by the ubiquitin-proteasome system (UPS), while α-synuclein aggregates and oligomers are preferentially cleared via the autophagy-lysosomal pathway (ALP). However, structural constraints of the proteasome and the inability of chaperones to efficiently unfold α-synuclein oligomers for degradation indicate the critical role of autophagy in mitigating the burden of aggregated α-synuclein [3-6].

Mutations and polymorphisms in the *SNCA* gene are implicated in familial and sporadic forms of PD, further exacerbating α-synuclein aggregation. *SNCA* gene duplication and point mutations, such as A53T, A30P, and E46K, increase the propensity of α-synuclein to misfold and form toxic oligomers and fibrils. These genetic alterations not only enhance α-synuclein aggregation but also disrupt normal pathways of cellular trafficking and degradation, leading to its accumulation [7,8]. The interplay between genetic mutations and impaired protein clearance underscores the potential of targeting aggregation-prone α-synuclein species through pathways such as autophagy to mitigate PD progression. Aggregated α-synuclein triggers autophagy, a crucial cellular breakdown pathway. During autophagy, cytosolic components, including aggregated proteins, are sequestered into double-membrane vesicles called autophagosomes. These then merge with lysosomes for degradation. Promoting autophagy may help remove α-synuclein aggregates and slow disease progression in PD [9].

**Figure 1.**
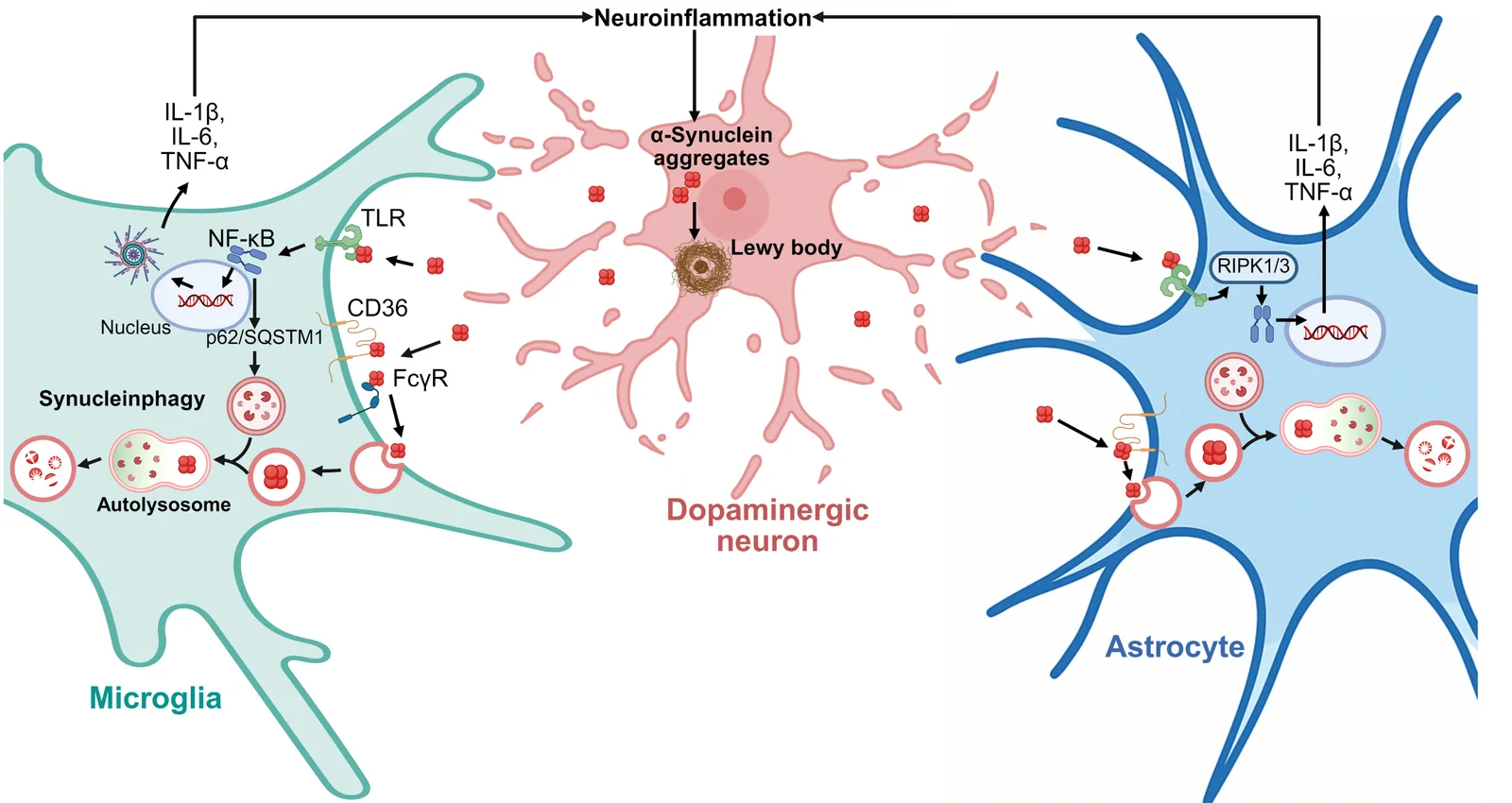
**Extracellular α-synuclein: a double-edged signal balancing neuro-inflammation and autophagic clearance**. Fibrillar α-synuclein released from degenerating dopaminergic neurons acts via two parallel mechanisms: (i) it activates TLR2/4-, CD36-, and FcγR-dependent pathways, leading to NF-κB and RIPK1/3 signaling in neighboring microglia and astrocytes, which promotes the secretion of IL-1β, IL-6, and TNF-α; and (ii) it is selectively targeted for degradation via p62/SQSTM1-mediated autophagy, in which α-synuclein is sequestered into autophagosomes and subsequently cleared through lysosomal fusion, a process known as ‘synucleinphagy’. This balance between pro-inflammatory signaling and degradative clearance shapes the neuroinflammatory milieu and ultimately influences neuronal survival. TLR: Toll-like receptor; NF-κB: Nuclear factor kappa B; RIPK: Receptor-interacting protein kinase; IL: Interleukin; TNF: Tumor necrosis factor; SQSTM: Sequestosome.

Autophagy is a carefully regulated cellular process that involves the degradation and recycling of damaged organelles, misfolded proteins, and other cytoplasmic components. Cellular stress or nutrient deprivation initiates the process by activating or inhibiting upstream regulators, such as 5’-AMP-activated protein kinase (AMPK) and mechanistic target of rapamycin complex 1 (mTORC1), leading to activation of the Unc-51 like autophagy activating kinase 1 (ULK1) complex. This triggers the formation of a membrane structure known as the phagophore, which is guided by the Beclin-1-VPS34 complex and other autophagy-related (ATG) proteins. The phagophore then expands, wrapping around its cargo to create a double-membraned vesicle known as the autophagosome. Two primary strategies have been explored to enhance autophagy for the clearance of α-synuclein aggregates. The first involves increasing autophagosome formation by targeting the upstream regulators of autophagy initiation [10]. Pharmacological agents such as metformin, rapamycin, nilotinib, and nicotinamide have been shown to effectively promote autophagy initiation. The second strategy focuses on facilitating autophagosome maturation. In these processes, the autophagosome fuses with the lysosome to form an autolysosome, allowing for the degradation and recycling of its contents. Once the autophagosome is fully formed and has enclosed damaged organelles or misfolded proteins, it is transported along microtubules toward the perinuclear region, where lysosomes are concentrated. This transport is regulated by motor proteins and small GTPases, such as Rab7. The fusion between the autophagosome and the lysosome is mediated by specific tethering factors and SNARE proteins, including Syntaxin 17 (STX17), synaptosome-associated protein 29 (SNAP29), and vesicle-associated membrane protein 8 (VAMP8). Upon fusion, lysosomal hydrolases degrade both the inner autophagosomal membrane and its cargo, breaking them down into basic biomolecules like amino acids, lipids, and sugars. These are then transported back into the cytoplasm for reuse. Small GTPases, such as RAB7 of the RAS oncogene family, and their effectors, such as FYVE and coiled-coil domain autophagy adaptor 1 (FYCO1), play critical roles in regulating these processes. Their potential for reducing α-synuclein pathology has been demonstrated in cellular and animal models of PD [11,12]. Additionally, misfolded or aggregated α-synuclein acts as a damage-associated molecular pattern (DAMP) recognized by innate immune receptors on microglia, such as Toll-like receptors (TLRs), particularly TLR2 and TLR4. Activation of these receptors triggers inflammatory signaling cascades, including the NF-κB pathway, promoting the release of pro-inflammatory cytokines, chemokines, and reactive oxygen species (ROS). In parallel, α-synuclein-induced neuroinflammation can engage receptor-interacting protein kinases RIPK1 and RIPK3, key mediators of necroptosis, a regulated form of inflammatory cell death. Activation of RIPK1/3 signaling contributes to microglial activation, cytokine release, and neuronal cell death, thereby amplifying the cycle of neurodegeneration and chronic inflammation characteristic of synucleinopathies [13,14] (Fig. 1).

Current pharmacological treatments for PD, including levodopa, monoamine oxidase B (MAOB) inhibitors, and dopamine agonists, provide symptomatic relief but are often associated with significant side effects, such as motor fluctuations and cognitive impairments. In this context, autophagy-enhancing agents, such as trehalose, isorhynchophylline, latrepirdine, and nilotinib, offer a promising therapeutic approach. These agents have demonstrated efficacy in promoting the degradation of mutant α-synuclein and alleviating pathological features in preclinical models of PD. Therefore, this review aims to explore recent advances in the clearance of α-synuclein aggregates through autophagy in depth. By examining molecular mechanisms, therapeutic strategies, and emerging agents targeting the ALP, we aim to highlight the potential of autophagy modulators as innovative treatments for PD.

## Defective autophagy in PD

Autophagy is a crucial process for maintaining cellular homeostasis and promoting cell differentiation and survival by degrading cytoplasmic elements, including proteins, lipids, nucleic acids, and damaged mitochondria. In lysosome-mediated autophagy, or macroautophagy, defective proteins or organelles are engulfed by a double-layered autophagosome, which then fuses with a lysosome to form an autophagolysosome. Autophagosome formation is primarily regulated by the inhibition and activation of mTOR and AMPK, respectively [15,16]. α-synuclein has a complex bidirectional relationship with autophagy; dysfunctional autophagy can lead to α-synuclein aggregation, and α-synuclein aggregation can interfere with autophagy, impairing the clearance of α-synuclein and other cellular substances [17,18]. Several genes associated with PD have a detrimental effect on autophagy, either by impairing autophagosome formation and maturation or lysosome function.

Leucine-rich repeat kinase 2 (*LRRK2*) is associated with both familial and sporadic PD. The function of LRRK2 is complex, with roles in various cellular processes, particularly neuronal function and maintenance. Mutations in *LRRK2* (G2019S and R1441C) and *Lrrk2* knockdown or knockout in mice impair autophagy in PD. LRRK2 inhibits autophagy by blocking phagophore biogenesis and autophagosome formation, leading to deficient autophagosome-lysosome fusion [19]. In chaperone-mediated autophagy (CMA), lysosome-associated membrane protein 2 splice variant A (LAMP2A) translocates substrates into the lysosome via the formation of a translocation complex by oligomerization. Mutated LRRK2 binds strongly to LAMP2A and inhibits its oligomerization, preventing the formation of the necessary translocation complex and blocking lysosomal degradation of α-synuclein and other substrates. This impaired autophagy leads to α-synuclein accumulation in neurons [20].

The glucocerebrosidase-beta (*GBA*) gene encodes the GCase lysosomal enzyme that hydrolyzes glucosyl-ceramide (GlcCer) to ceramide and glucose, essential for maintaining cellular homeostasis and proper lysosomal function. An estimated 5%-10% of patients with PD have *GBA* mutations, with N370S and L444P the most common, making them major genetic risk factors [21]. Mutations in GBA cause its loss of function, leading to deficient GCase activity and GlcCer accumulation in the lysosome. Consequently, α-synuclein cannot enter the lysosome for degradation, increasing its aggregation [20]. However, α-synuclein aggregation can also impair GCase activity by interfering with GCase trafficking from the endoplasmic reticulum to the lysosome via the Golgi body [22].

Membrane expansion for autophagosome formation is a complex and poorly understood process, for which the autophagy-related protein ATG9 is crucial. α-synuclein overexpression and aggregation cause the mislocalization of ATG9 by RAB1A, a member of the RAS oncogene family, impairing autophagosome formation [23,16]. Autophagosome-lysosome fusion is another key event in the degradation of unwanted molecules via autophagy. SNAP29 is a vesicle SNARE protein that mediates the fusion of autophagosomes with lysosomes. Overexpression of α-synuclein impairs SNAP29-mediated autophagosome-lysosome fusion [24].

Mutations in *SNCA* can cause familial early-onset PD. Studies have shown that mutations such as A30P, A53T, and E46K result in rapid fibril formation and variations in their structure [25,26]. Both wild-type (WT) and mutated α-synuclein impair the autophagy pathway. Zinc finger with Krüppel-associated box (KRAB) and SCAN domains 3 (ZKSCAN3/ZNF306) belongs to a family of zinc-finger transcription factors that transcriptionally repress autophagy [27]. The α-synuclein^A30P^ mutant is reported to inhibit autophagy by affecting JUN N-terminal kinase (JNK) signaling, thereby enhancing ZKSCAN3 activity [28]. Variations in fibril structure and properties caused by mutations can impact autophagy through diverse mechanisms. It is therefore essential to finely modulate the pathways involved in their clearance. However, few studies have examined the effects of mutations on fibril clearance, warranting further exploration of this topic.

**Figure 2.**
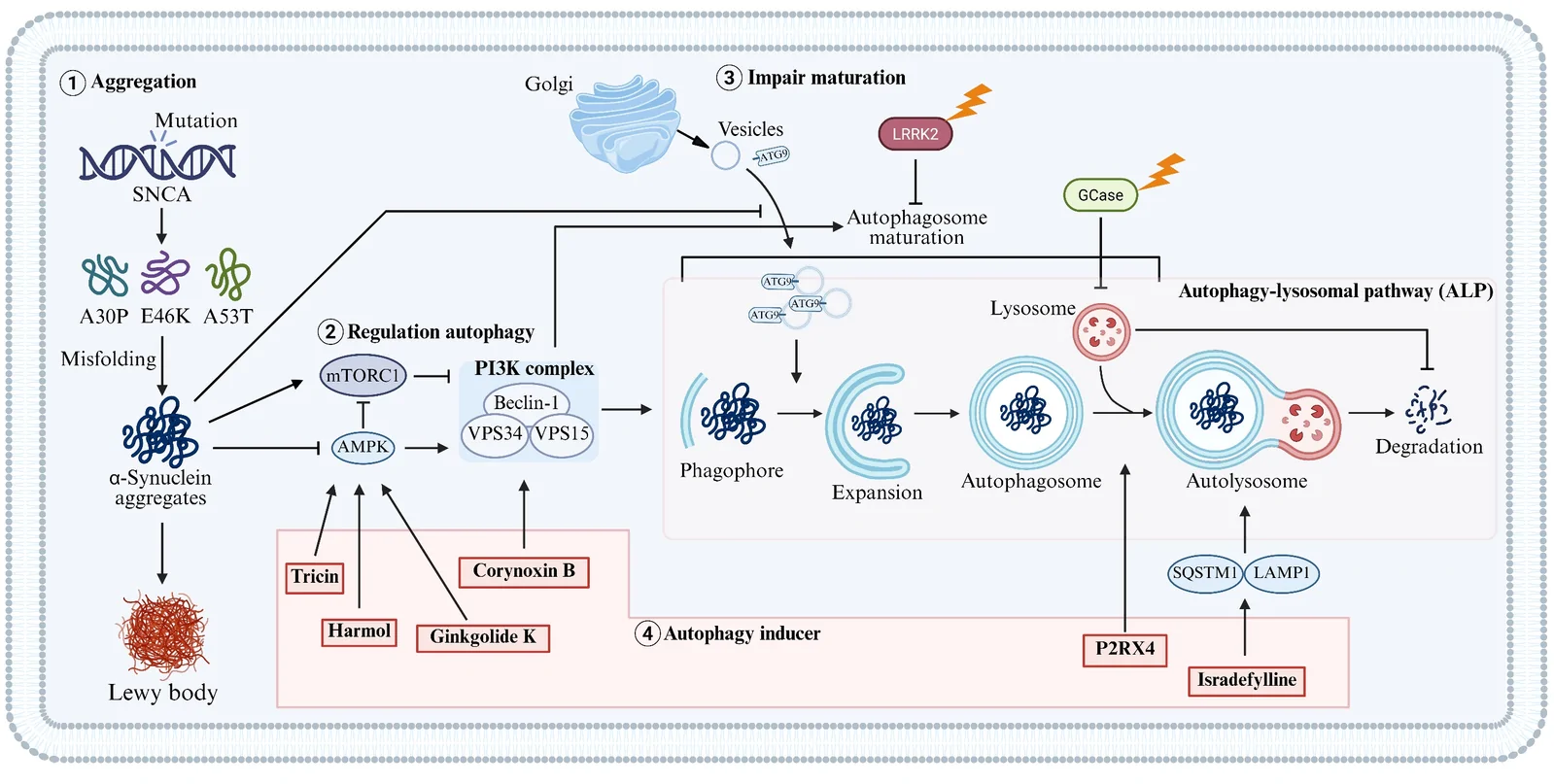
**Schematic overview of autophagy-mediated clearance of aggregated α-synuclein in PD**. (1) Pathogenic α-synuclein mutations (A30P, E46K, A53T) promote misfolding and aggregation, ultimately leading to Lewy body formation. (2) The diagram illustrates key regulatory components of the autophagy-lysosomal pathway (ALP), including AMPK, mTORC1, and the class III PI3K complex (Beclin-1, VPS34, VPS15), which are critical for phagophore formation and autophagosome maturation. (3) Aggregated α-synuclein disrupts ATG9 vesicle trafficking from the Golgi, impairing phagophore initiation, while dysfunction of LRRK2 and reduced GCase activity independently impair autophagosome maturation and lysosomal degradation, contributing to global autophagic dysfunction. (4) Several small-molecule modulators (Tricin, Ginkgolide K, Corynoxin B, Harmol) and pathways involving P2RX4, SQSTM1, and LAMP1 are shown to restore autophagic flux and promote α-synuclein degradation, potentially alleviating neurodegeneration. PD: Parkinson’s disease; AMPK: AMP-activated protein kinase; mTORC1: Mammalian target of rapamycin complex 1; PI3K: Phosphoinositide 3-kinase; GCase: Glucocerebrosidase; VPS: Vacuolar protein-sorting; ATG: Autophagy-related protein; LRRK: Leucine-rich repeat kinase.

## Small molecules activate autophagy and eliminate toxic protein aggregates

Autophagy inducers are chemicals or interventions that increase ALP activity, facilitating the degradation and removal of accumulated proteins. These inducers promote the autophagic breakdown of α-synuclein, preventing harmful aggregation, reducing cellular stress, and protecting susceptible neurons. Advances in the understanding of autophagy mechanisms and the development of tailored autophagy inducers have opened new avenues for therapeutic interventions in PD. This review examines how autophagy inducers can facilitate the clearance of accumulated α-synuclein, including their mechanisms of action, therapeutic efficacy, and implications for PD development.

Recent advances in autophagy-inducing small molecules highlight promising therapeutic avenues for PD. These molecules act through diverse mechanisms to target autophagy-related proteins, lysosomal biogenesis, and CMA activation, providing potential alternatives to conventional symptomatic treatments. Small molecules that modulate autophagy have shown great promise in facilitating the clearance of α-synuclein aggregates through promoting autophagosome formation or enhancing autophagosome maturation and lysosomal degradation (Table 1, Fig. 2).

**Table 1:** Small-molecule autophagy inducers that help to clear α-synuclein aggregates.

Compound	Study model	Dose	Duration of treatment	Therapeutic effects	Refs.
***In Vitro* study**					
**Trehalose, lactulose or, melibiose**	α-synuclein-GFP SH-SY5Y cells	100 μM	8 hours	Increased LC3-II/LC3-I ratio, *NRF-2* expression, and α-synuclein aggregates were inhibited and cleared.	[32]
**Corynoxin B**	N2a cells, PC-12 cells	20 mM/L,	6 hours	Enhanced BECN1/VPS34 complex activity, promoted the interaction between BECN1 and HMGB1/2 which induced autophagy for α-synuclein^WT^ and α-synuclein^A53T^ aggregate clearance.	[37]
**Ginkgolide K**	SH-SY5Y cells	30 μg/mL	24 hours	Activated the LC-II/class-III PI3K pathway and promoted α-synuclein^A53T^ clearance.	[41]
**Maysin**	SH-SY5Y cells	5 μM	24 hours	Reduced RPS6 expression, increased the LC3-II/LC3-I ratio, and enhanced autophagosome formation to clear protein aggregates.	[43]
**Tricin**	Tet-on α-synuclein^A53T^ inducible PC-12 cells SH-SY5Y cells	40-100 μM	24 hours	Activated the AMPK-p70s6K signaling pathway and ATG7-dependent autophagy and enhanced α-synuclein clearance in both cellular models.	[44]
**Celastrol**	MoDCs	0.25 µM	1 hour	Upregulated *Rab5* and *Rab7* expression, increased *Becn1* expression, reduced *p62* expression, and elevated LC3 ratio facilitated the trafficking of α-synuclein towards the lysosome and promoted the clearance of fibrillar proteins.	[47]
**Berberine, coptisine, and palmatine**	α-synuclein^A53T^-GFP SH-SY5Y cells	10 μM	8 hours	Increased TFEB expression and the LC3-II/LC3-I ratio induced autophagy and inhibited α-synuclein^A53T^ aggregation.	[48]
**GM1 ganglioside**	PC-12 (α-synuclein^A53T^) cells	40 μg/mL	24 hours	Phosphorylation of ATG13 and ULK1 enhanced SCNA^A53T^ degradation.	[49]
**Albendazole**	SH-SY5Y cells	10 μM	8 hours	Induced lysosomal clustering through a JIP4/TRPML1-dependent mechanism and autophagy-dependent degradation of Triton-X-insoluble aggregates.	[53]
**Mc1568**	SH-SY5Y cells	0-10 μM	2 hours	HDAC4 inhibition induced autophagy.	[59]
**AGK2**	SH-SY5Y cells	5 mM	24 hours	Increased phosphorylation of P70S6K, decreased total 4EBP1, and clearance of α-synuclein aggregates from the cell enhanced cell survival.	[60]
**Harmol**	Tet-on α-synuclein^A53T^ inducible PC-12 cells	30 μM	6-24 hours	Phosphorylation of ULK1 at Ser757 activated the AMPK/mTOR/TFEB pathway, promoting autophagy and lysosomal biogenesis.	[64]
**Dihydromyricetin (DHM) and salvianolic acid B (Sal B)**	H4 neuroglioma cells expressing SynT-aggregation	DHM (10 μM), Sal B (50 μM)	16 hours	Increased LAMP1 and LAMP2A levels and degraded α-synuclein aggregates.	[73]
***In Vivo* study**					
**Corynoxin B**	*Prp*-α-synuclein α-synuclein^A53T^ transgenic mice	20 mg/kg, intraperitoneal injection	Daily for one month	Cleared the insoluble or aggregated α-synuclein^WT^ and α-synuclein^A53T^ and improved behavioral performances.	[37]
**Istradefylline (KW6002)**	Male C57BL/6 mice	5 mg/kg/body weight (b.w.), intraperitoneal injection	Every other day for 1 month	Regulating SQSTM1 and LAMP2A expression cleared α-synuclein^A53T^ protofibrils from the mouse frontal cortex.	[63]
**Harmol**	α-synuclein^A53T^ mice	40 mg/kg/b.w, oral gavage	Twice a day, Daily for 1 month	Activated the AMPK/mTOR/TFEB pathway, promoting autophagy leading to the clearance of aggregated protein in substantia nigra	[64]
**Ferruginol**	Transgenic α-synuclein^A53T^ C57BL/6 mice	50 or 100 mg/kg/b.w., intraperitoneal injection	Daily for 21 days	Increased BECN1/LC3-I/II ratio attenuated α-synuclein aggregation and altered behavioral deficits.	[65]
**HSEA-P1, P2**	*C. elegans* (NL5901 strain)	10 μg/mL, dropped in nematode growth media agar plate	Every other day for 9 days	Regulated *bec-1* (BECN1), *lgg-1* (LC3), and *atg-7* (ATG7) and degraded α-synuclein aggregates.	[67]
**Piperine**	*Thy1*-α-synuclein transgenic mice expressing human α-synuclein	25, 50, and 100 mg/kg/b.w., oral administration	Daily for 6 weeks	Activated P2RX4-mediated autophagy, degraded aggregated α-synuclein *in vivo*	[72]
**Dihydromyricetin (DHM) and salvianolic acid B (Sal B)**	Transgenic mice expressing human α-synuclein^WT^	10mg/kg/b.w./day, intraperitoneal injection	Daily for 2 weeks	Increased LAMP1 and LAMP2A levels and degraded α-synuclein aggregates.	[73]

Abbreviations: SNCA: Synuclein alpha; α-syn: Alpha synuclein; GFP: Green fluorescence protein; LC3: Microtubule-associated protein 1A/1B-light chain 3; Nrf-2: Nuclear factor erythroid 2-related factor 2; N2a cells: Neuro2a cells (mouse neuroblastoma); PC-12: VPS34: Vacuolar protein-sorting 34; HMGB1/2: High Mobility Group Box 1 and 2 protein; PI3K: Phosphoinositide 3-kinase; AMPK: AMP-activated protein kinase; ATG: Autophagy-related protein; Rab: Ras-associated binding; TFEB: Transcription Factor EB; ULK: Unc-51-like kinase; HDAC: Histone deacetylase; SQSTM: Sequestosome; LAMP: Lysosome-associated membrane protein.

Trehalose, a plant disaccharide, has proven effective against aggregate-prone NDDs [29-31]. Lactulose and melibiose, two indigestible analogs of trehalose, were found to exhibit promising effects of α-synuclein aggregation inhibition and autophagy induction. In human neuroblastoma SH-SY5Y cells, induced expression of SNCA-GFP (green fluorescent protein), combined with the addition of α-synuclein fibrils over six days, reduced the microtubule-associated protein 1 light chain 3 (LC3-II/LC3-I) ratio to 57% (p < 0.001) compared to control cells (100%). Meanwhile, treatment with trehalose, lactulose, or melibiose (100 μM) significantly increased the ratio to 86%-90% (p = 0.012-0.005) relative to the fibril-treated cells [32]. The primary signaling complex needed for autophagosome formation and maturation is the phosphatidylinositol 3-kinase III (PI3KC3) complex, which consists of vacuolar protein sorting 34 (VPS34), vacuolar protein sorting 15 (VPS15), and beclin 1 (BECN1). The PI3KC3 complex synthesizes phosphatidylinositol 3-phosphate (PI3P); PI3P promotes the recruitment of further autophagic components and encourages the autophagosome’s double membrane to expand and the lysosome to fuse with the autophagosome [33-35]. α-synuclein^WT^ and α-synuclein^A53T^ bind to high mobility group box 1 (HMGB1), inhibiting its translocation to the cytosol and thereby preventing its interaction with BECN1, impairing autophagy [36]. Corynoxin B (Cory-B) is an alkaloid from *Uncaria rhynchophylla* (Miq.) Jacks that produced promising results in enhancing α-synuclein clearance from cells. Treating rat pheochromocytoma PC-12 cells with 20 mM/L of Cory-B significantly activated the PI3KC3 complex and increased the interaction between HMGB1/2 and BECN1. In mouse neuroblastoma N2a cells, Cory-B bound directly to HMGB1/2. Treating transgenic mice with 20 mg/kg Cory-B promoted the clearance of α-synuclein^WT^ and α-synuclein^A53T^ and improved behavioral outcomes [37].

Ginkgolide K, a compound isolated from *Ginkgo biloba* leaves, exhibits neuroprotective activity in PD [38,39]. In a cellular PD model, ginkgolide K induced autophagy through the AMPK/mTOR/ULK1 signaling pathway [40]. In SH-SY5Y cells overexpressing α-synuclein^A53T^, ginkgolide K at 30 mg/mL cleared mutated α-synuclein from cells by 50-fold through the lipidated light chain 3 (LC3-II)/PI3K pathway [41]. Autophagy was also induced by maysin, a C-glycosyl flavone (rhamnosyl-6-C-(4-ketofucosyl)-5,7,3′,4′ tetrahydroxyflavone), isolated from corn silk (*Stigma maydis*). Treating SH-SY5Y cells with 5 mM maysin significantly reduced ribosomal protein S6 (RPS6) phosphorylation-a crucial downstream substrate of the primary suppressive regulator of mTOR [42]-thus inducing autophagy. The increased LC3-I/LC3-II ratio confirmed autophagosome formation [43]. Tricin, a flavonoid, induces autophagy in PC-12 cells by activating AMPK through the regulation of the AMPK-p70S6 kinase (p70s6K) signaling cascade, clearing α-synuclein^A53T^ and enhancing autophagy-related 7 (ATG7)-dependent autophagy [44].

In healthy individuals, dendritic cells (DCs) are either absent or found in low numbers in the central nervous system (CNS). In the early stages of PD development, peripheral DCs cross the blood-brain barrier (BBB) and react to α-synuclein aggregates, causing neuro-inflammation. Ng et al. demonstrated that fibrillar α-synuclein impaired autophagy in monocyte-derived DCs (MoDCs). Treating cells with celastrol (0.25 µM), isolated from *Tripterygium wilfordii* Hook F., induced autophagosome formation, decreased p62 expression, and increased the LC3-I/LC3II ratio. In addition, this treatment colocalized fibrillar α-synuclein with autophagy markers, indicating that celastrol contributes to the clearance of α-synuclein fibrils from MoDCs [45-47]. *Coptis chinensis* rhizome extract, widely used in traditional Chinese medicine, contains alkaloids including berberine, coptisine, and palmatine. Treating SH-SY5Y cells containing GFP-labeled α-synuclein^A53T^ using the extract (100 mg/mL) or its constituents (10 mM) significantly induced autophagy via the transcription factor EB (TFEB)-mediated pathway and reduced the aggregated protein by 70 percent [48]. GM1 ganglioside, a brain ganglioside that modulates neuroplasticity and releases neurotransmitters, ameliorated α-synuclein accumulation and reduced neurotoxicity by inducing α-synuclein clearance in 1-methyl-4-phenylpyridinium (MPP^+^)-induced SH-SH5Y cells. Treating PC-12 cells containing α-synuclein^A53T^ with 40 mg/mL of GM1 ganglioside induced autophagy by regulating the levels of autophagy-related 13 (ATG13), phosphorylated (p)-ATG13 (Ser355), ULK1, and p-ULK1 (Ser555). In a 1-methyl-4-phenyl-1,2,3,6-tetrahydropyridine (MPTP; 30 mg/kg)-induced mouse model of PD, treatment with 50 mg/kg GM1 ganglioside improved motor and behavioral deficits [49].

Recent research indicates that lysosomal clustering around the microtubule-organizing center (MTOC) is vital for regulating autophagic flux. This arrangement increases the fusion of lysosomes and autophagosomes while inhibiting mTORC1, thus increasing autophagy [50,51]. PD patients form α-synuclein aggregates around the MTOC, indicating that MTOC-centered autophagy may contribute to clearing them [52]. Based on this hypothesis, Date et al. examined multiple compounds that promote lysosomal clustering near MTOCs, potentially reducing the distance between lysosomes, autophagosomes, and degradation substrates. Among teniposide, amsacrine, albendazole, etoposide, and mebendazole, 10 μM albendazole enhanced lysosomal accumulation. Albendazole degraded α-synuclein aggregates via JNK-interacting protein 4 (JIP4)-mediated lysosomal positioning, and by phosphorylating the JIP4/mucolipin TRP cation channel 1 (MCOLN1/TRPML1)/ALG2 alpha-1,3/1,6-mannosyl-transferase (ALG2) complex [53,54].

Histone deacetylases (HDACs) may regulate autophagy by modulating the acetylation levels of specific histone sites and autophagy-related ATG proteins. Acetylation of histone H3 at lysine 56 (H3K56ac) and histone H4 at lysine 16 (H4K16ac) is strongly associated with autophagy pathway regulation [55,56]. HDAC4 is abundant in brain tissue and reportedly colocalizes with α-synuclein. Several HDAC inhibitors, including trichostatin, butyrate, benzyl butyrate, and valproate, have been investigated for the treatment of PD [57,58]. Wang et al. found that Mc1568, a selective HDAC4 inhibitor, induced autophagy in a rotenone-induced SH-SY5Y cell model of PD. Treatment with 10 mM Mc1568 increased the LC3-I/II ratio and reduced aggregated protein levels by 50-fold [59].

In macroautophagy, sequestosome 1 (SQSTM1/p62) and lysosomal-associated membrane protein 1 (LAMP1) play crucial roles in protein degradation through autolysosome formation [61,62]. KW6002, also known as istradefylline, is an adenosine A2A receptor inhibitor that was tested for its autophagic efficiency in α-synuclein clearance. α-synuclein^A53T^ protofibrils were injected into male C57BL/6 mice and transfected mouse hippocampal HT-22 cells. KW6002 regulated the expressions of SQSTM1 and LAMP1, influencing autolysosome formation in the prefrontal cortex neurons of treated mice. In HT-22 cells, KW6002 modulated the expression of autophagy-related proteins LC3-I/II, SQSTM1, LAMP2A, and heat shock cognate protein 70 (HSC70). Interestingly, KW6002 also induced molecular CMA by upregulating the heat shock protein 70 (HSP70) family, which recognizes the α-synuclein KFERQ motif and transports it to the autolysosome for degradation, clearing the aggregated protein by 20 ± 4.5 % [63]. Recently, treatment of PD mice (α-synuclein^A53T^) and PC-12 cells with 40 mg/kg and 30 mM, respectively, of the autophagy inducer harmol was reported to activate the AMPK/mTOR/TFEB pathway to induce autophagosome-lysosome biogenesis. Treating PC-12 cells with harmol also induced ULK1 phosphorylation at Ser757, which is involved in autophagosome formation [64].

Activating the autophagy initiator downstream protein is a promising strategy for inducing autophagy. Ferruginol is a natural phenol isolated from the needles of the redwood *Sequoia sempervirens* and the immature cones of *Chamaecyparis lawsoniana*. Treating α-synuclein^A53T^ transgenic C57BL/6 mice with 50-100 mg/kg of ferruginol induced lysosome-mediated autophagy and reduced protein aggregate levels [65]. *Caenorhabditis elegans* is a popular model of PD with several biological advantages, including the ease of visualizing its eight well-characterized dopaminergic neurons and the ability to monitor behaviors associated with their function [66]. Treatment of a *C. elegans* PD model (NL5901 [pkIs2386(Punc-54::α-synuclein::YFP + unc-119)]) with HSEA-P1 and -P2-palmitic acids isolated from the sea cucumber *Holothuria scabra* Jaeger-reduced α-synuclein aggregation in muscle cells. The autophagy-related genes *bec-1* (BECN1), *lgg-1* (LC3), and *atg-7* (ATG7) were necessary for this degradation, and their expression was regulated by these compounds [67].

Purinergic receptor P2RX4, a member of the P2X receptor family, acts as a cation-permeable ion channel activated by the binding of extracellular ATP. Typically found on the plasma membrane, P2RX4 can be internalized into late endosomes, lysosomes, or lysosome-related organelles. Under normal conditions, P2RX4 is stable within lysosomes; however, its activation induces lysosomal Ca^2+^ release, promoting membrane fusion with autophagosomes. These insights suggest that P2RX4 could be a therapeutic target for modulating the ALP [68-71]. At 25 mM, piperine, an alkaloid isolated from *Piper longum* L., induced ALP activation and degraded α-synuclein aggregates in human neuroblastoma SK-N-SH cells. In a PD mouse model, treatment with 100 mg/kg piperine induced autophagy by activating P2RX4 [72].

## Immune system and α-synuclein clearance

Immune cell activation in response to α-synuclein aggregation causes neuroinflammation, contributing to PD progression. α-synuclein aggregates stimulate brain-resident glial cells, including microglia and astroglia, inducing neuroinflammation [74-76]. Uncontrolled microglial activation leads to higher expression of major histocompatibility complex antigens and the secretion of pro- and anti-inflammatory cytokines. As PD progresses, microglial activation causes the release of inflammatory cytokines such as tumor necrosis factor alpha (TNF-a), interleukins 1 beta (IL1b) and 6 (IL6), and interferon-gamma (IFN-g) in the substantia nigra [77,78]. Evidence from both *in vitro* (SH-SY5Y cells) and clinical experiments suggests that α-synuclein aggregation also induces neuroinflammation through the interleukin 6 cytokine family signal transducer-antisense (IL6ST-AS)/signal transducer and activator of transcription 3 (STAT3)/hypoxia-inducible factor 1 subunit alpha (HIF1-a) axis [79]. Abnormal α-synuclein aggregation also activates the NLR family pyrin domain containing 3 (NLRP3) inflammasome in microglia via TLRs. This leads to nuclear factor kappa B (NF-κB) translocation, pro-inflammatory cytokine release, mitochondrial dysfunction, and subsequent damage to dopaminergic neurons [80].

Besides initiating the inflammatory response, microglial cells play a direct role in clearing α-synuclein aggregates. Microglia are brain-resident glial cells that regulate various functions in CNS, including the maintenance of neuronal networks and injury repair [81]. Microglial autophagy is also essential for suppressing neuroinflammation in the CNS. Extracellular α-synuclein can inhibit microglial autophagy by dysregulating the TLR4-dependent p38 mitogen-activated protein kinase (MAPK) and protein kinase B (AKT)-mTOR signaling cascade [82]. Microglia clear extracellular α-synuclein via phagocytosis or autophagy by recognizing α-synuclein aggregates with TLRs, Fc-gamma receptors (FcγRs), and scavenger receptors [83]. Once α-synuclein is recognized, intracellular signaling pathways become activated and lead to phagosome formation, which fuse with lysosomes to form phagolysosomes. Several lysosomal enzymes subsequently degrade the α-synuclein aggregates [84,85]. Choi et al. demonstrated the importance of TLR4 in inducing transcriptional upregulation of p62/SQSTM1 in both primary microglial cells and mouse models. p62 induction initiates autophagy, inducing microglia to clear α-synuclein aggregates via TLR4/NF-κB/p62-mediated synucleinopathy [86,87]. In addition, Rocha et al. showed that knocking out microglial NF-κB/IKK_2_ inhibited p62/SQSTM1-dependent autophagy and enhanced α-synuclein aggregation in a rotenone mouse model [88], suggesting that microglia are important in clearing accumulated α-synuclein.

Alongside microglia, astrocytes also contribute to neuroinflammation and the clearance of extracellular α-synuclein. α-synuclein triggers astrocyte activation, exacerbating neuroinflammation and neurodegeneration [89,90]. α-synuclein preformed fibrils (PFFs) activate human midbrain astrocytes, causing inflammation, impaired phagocytosis, and neurotoxic effects via receptor-interacting serine/threonine kinase 1 (RIPK1) and 3 (RIPK3)-dependent NF-κB signaling. This observation revealed a novel role for α-synuclein in promoting neurotoxic astrocyte activation and cell death-independent functions of RIPKs in neuroinflammation and parkinsonian neurodegeneration [91]. TLR2 activation in astrocytes exacerbates α-synuclein aggregation and propagation by impairing the ALP, contributing to neuronal cell death [92]. Despite their potentially detrimental effects, astroglia also contribute to neuroprotection by facilitating the clearance of α-synuclein aggregates via mechanisms such as phagocytosis, lysosomal degradation, and extracellular proteolysis [93,94]. Tsunemi et al. demonstrated that astrocytes internalize α-synuclein, with a higher rate of lysosomal degradation than in neurons. They also found that coculturing neuronal cells with astrocytes reduced α-synuclein accumulation within neurons, limiting transfer between them [95]. α-synuclein was degraded through the ALP in astrocytes, and this clearance was enhanced by ginkgolide K [96]. Recently, Yang et al. demonstrated that astrocytes enhance neuronal autophagic clearance of α-synuclein through paracrine signaling, indicated by the colocalization of p-α-synuclein (Ser129) with autolysosomes. They also showed that transplanting ventral midbrain astrocytes into a PD mouse model reduced α-synuclein pathology and neuron degeneration [97]. These findings suggest the importance of microglia and astrocytes in clearing α-synuclein aggregates.

## Immunotherapy

### Active immunotherapy

Active immunity is the process by which a specific antigen stimulates the immune system to produce antibodies and immune cells [98]. It can occur naturally after infection or artificially via immunization. In PD, active immunization aims to train the immune system to recognize and target pathological forms of α-synuclein. By generating a sustained immune response, active immunization holds promise for reducing toxic α-synuclein accumulation, slowing PD progression, and offering a long-term therapeutic benefit [99] (Table 2). Studies have investigated active immunization against α-synuclein using transgenic mouse models to assess vaccine effectiveness and safety. An α-synuclein vaccine was shown to notably reduce α-synuclein pathology, characterized by reduced accumulation of α-synuclein aggregates and decreased neuronal degeneration [100-102]. AFFITOPE (AFF) vaccines are peptide-based and designed to stimulate the production of antibodies against α-synuclein [103,104]. Lemos et al. demonstrated that either alone or in combination, AFF PD03 and Anle138b improved gait deficits and preserved nigral dopaminergic neurons in a proteolipid protein (*Plp*)-α-synuclein mouse model; these effects were accompanied by reduced α-synuclein oligomers, decreased microglial activation, and lower immunoglobulin G (IgG) binding, ultimately slowing neurodegeneration. Clearance is believed to be mediated by microglia, resulting in increased expression of anti-inflammatory cytokines [105].

Based on promising preclinical findings, two Phase I clinical trials have examined the AFF PD01A and PD03A vaccines, assessing safety and tolerability in patients with PD and multiple system atrophy (MSA). The first, a randomized parallel-group Phase I trial, enrolled 32 patients with early-stage PD to evaluate vaccine effects. PD01A and PD03A were both safe and well-tolerated, and PD01A triggered a rapid and long-lasting antibody response [106]. The other trial assessed the safety, tolerability, and immunological activity of PD03A in 36 patients with early-stage PD randomized to receive 15 μg or 75 μg of PD03A or placebo over 52 weeks. Of these 36 patients, 35 completed all five immunizations. While all patients experienced adverse events, most were mild and unrelated to the treatment, with injection site reactions being the most common. A strong IgG antibody response was observed, peaking at week 12 and remaining significantly elevated in both active groups compared to the placebo group from weeks 8 to 52. The favorable safety profile and immune response support the further development of PD03A as an active immunotherapy for PD [107].

UB-312 is a synthetic peptide-based vaccine formulated to overcome immune tolerance and generate a humoral antibody response against oligomeric and fibrillary forms of α-synuclein. One study assessed the effect of active immunization against α-synuclein using the UB-312 vaccine in the thymus cell antigen 1 theta (*Thy1*)-α-synuclein/15 mouse model of PD. Young transgenic and WT mice underwent a six-week immunization regimen followed by a nine-week observation period. UB-312 immunization specifically targeted and attenuated oligomeric α-synuclein levels within the cerebral environment by 28%-50%. However, no statistically significant reduction in monomeric α-synuclein levels was observed, suggesting that UB-312 does not promote the oligomer disassembly into monomers but instead promotes their clearance from the CNS. However, this study did not directly discuss the clearance of oligomers through autophagy [108]. A randomized placebo-controlled Phase I clinical trial evaluated the safety, tolerability, and immunogenicity of UB-312 in 50 healthy participants who received three intramuscular injections at varying doses, or placebo. UB-312 was found to be generally safe, well-tolerated, and immunogenic, with a strong antibody response, including the ability to cross the BBB [109]. However, challenges remain, such as immune tolerance to self-proteins and effective antibody delivery across the BBB, which must be addressed for effective therapeutic outcomes [110].

### Passive immunotherapy

Passive immunity refers to the production of IgG antibodies to protect against infections. It can be natural, lasting weeks to months, or acquired through the injection of concentrated immune serum. Passive immunization for NDDs involves the administration of externally engineered antibodies targeting specific a-synuclein domains, mimicking the action of endogenous IgG. In PD treatment, these antibodies bind to extracellular α-synuclein to prevent its spread [98,111,112] (Table 2).

**Table 2:** Preclinical and Clinical studies of active and passive immunotherapy for α-synuclein clearance.

Type	Peptide	Study model	Dose	Duration of treatment	Target site	Therapeutic effects	Refs.
Active	PD03 and Anle138b	PLP α-Syn mice	PD03 = subcutaneous injection (200 μl each), Anle138b = food pellets containing 0.6 g compound/kg of food	5 months	C-terminus of α-Syn	Reduced α-Syn oligomer level and microglial activation	[105]
	**UB-312**	Thy1SNCA/ 15 mice	40 μg per injection	15 months	C-terminus of α-Syn	The level of α-Syn was reduced	[108]
Passive	Prasinezumab (PRX002) Phase I	Healthy individuals	0.3, 1, 3, 10, or 30 mg/kg, intravenous infusion	a single dose	C-terminus of α-Syn	Reduced free serum α-Syn	[114]
	**Phase II**	PD patients	1,500 or 4,500 mg	3 intravenous infusions once every 28 days	C-terminus of α-Syn	The result is expected to be published in 2026	[115]
	**Phase II**	PD patients	1,500 or 4,500 mg	intravenous infusions once every 4 weeks for 52 weeks	C-terminus of α-Syn	Much slower worsening of motor symptoms compared to the control group	[116]
	**BIIB054 (Cinpanemab)**	α-synA53T (M83), BAC-α-synWT, BAC-α-synA53T mice	30 mg/kg	intravenous infusions, weekly for 180 days	N-terminus (residues 1-10) of α-synuclein	Decreased α-Syn spreading and improved motor performance	[117]
	**Phase I**	Healthy individuals & PD patients (NCT02459886)	15 or 45 mg/kg	Single IV dose, 16 weeks	N-terminus (residues 1-10) of α-synuclein	Showed selectivity for the aggregates, found safe and crossed BBB	[118]
	**Phase II**	Phase II (SPARK): Mild to moderate PD (NCT03318523)	250, 1,250, or 3,500 mg	Every 4 weeks for two years	N-terminus (residues 1-10) of α-synuclein	Discontinued (did not meet primary or secondary endpoints)	[119]
	MEDI1341	Sprague Dawley Rats	3 mg/kg to 100 mg/kg	Intravenous, for 13 weeks	C-terminus of α-Syn	Reduced CSF fluid α-Syn levels 81.5 ± 4.6% at the top dose of 100 mg/kg	[120]
		**Cynomolgus monkeys**	25, 75, and 150 mg/kg			Decreased CSF fluid α-Syn levels 88 ± 1.8% at the top dose of 150 mg/kg	
		**Lentivirus-α-synuclein-expressed mice**	20 mg/kg	Intraperitoneal (IP), for 13 weeks		Decreased α-Syn propagation along axons	
	**ABBV-0805 (BAN0805)**	Thy1-A30P and α-synA53T mice	0.1, 1, 10, and 20 mg/kg	Intravenous, Intraperitoneal, 2-4 months	C-terminus of oligomers & protofibrils	Decreased soluble and insoluble α-Syn Increased mean survival of A30P mice	[110, 121]
	**Lu AF82422**	cynomolgus monkey	100-600 mg/kg	Intravenous, 56 days	C-terminus	Reduced α-Syn in plasma and CSF	[122]
	**DR5-12D**	NKAα1+/+ and NKAα1+/- mice	30 mg/kg	Intraperitoneal for 83 days	-	DR5-12D activates NKAα1-dependent autophagy by enhancing signaling through the AMPK/mTOR/ULK1 pathway and clears aggregated protein	[123]

Abbreviations: NKAα: Na+/K+-ATPase- alpha subunit

The first Phase 1 clinical trial examining the anti-α-synuclein antibody PRX002 involved 40 healthy adults who received a single intravenous infusion of 0.3, 1, 3, 10, or 30 mg/kg. PRX002 effectively lowered free serum α-synuclein levels, suggesting clearance from the peripheral circulation. This reduction occurred quickly, within an hour of administration, and was dose-dependent. These findings suggest that clearance may be mediated by microglial activity and neuronal autophagy. PRX002 was also found to be safe and well-tolerated [113]. Later, the PASADENA trial, a two-year Phase II clinical trial by Roche, evaluated the efficacy of prasinezumab (1,500 or 4,500 mg) in patients with early PD. Its primary aim was to assess motor function improvement using the Movement Disorder Society-Unified Parkinson’s Disease Rating Scale. However, the latest 52-week report indicated no significant difference between the treatment and placebo groups. The trial is expected to conclude in 2026 [114, 115]. Another Roche clinical trial, PADOVA Phase IIB, involves 575 patients with more advanced PD symptoms than those in the PASADENA trial. These patients are on stable dopamine agonists and receive monthly injections of prasinezumab or a placebo for 18 months. The trial’s primary outcome is motor progression, with secondary outcomes of motor function and infusion reaction, the most common side effect. Prasinezumab treatment slowed motor progression in PD over 4 years, with delayed- and early-start groups showing 51%-65% slower decline in MDS-UPDRS Part III (OFF), 94%-118% slower decline in Part III (ON), and 40%-48% slower decline in Part II scores compared to an external comparator. These findings suggest a sustained long-term benefit, but must be confirmed in further studies [116].

Biogen’s monoclonal antibody (mAb), BIIB054, is a recombinant human IgG1 antibody produced by the memory B cells of older adults without neurological disorders. It binds to the N-terminus (residues 1-10) of α-synuclein. In a preclinical study, 30 mg/kg BIIB054 was administered to transgenic α-synuclein^A53T^ (M83), transgenic bacterial artificial chromosome (BAC)-α-synuclein^WT^, and transgenic BAC-α-synuclein^A53T^ mice via intraperitoneal injection. This treatment decreased α-synuclein spreading by 30%, dopamine transporter (DAT) density loss in dopaminergic terminals by 20%, and improved behavioral performance in the wire-hanging test by 50% [117]. Due to the promising effects, a randomized, double-blind, placebo-controlled clinical trial (NCT02459886) evaluated the safety and pharmacokinetics of BIIB054 (cinpanemab) in healthy individuals and patients with mild to moderate PD. Participants received a single dose (15 or 45 mg/kg) of BIIB054 or placebo and were assessed through clinical, neuroimaging, electrocardiography (ECG), and laboratory measures for 16 weeks. BIIB054 exhibited a favorable safety profile, crossed the BBB, and was more selective for α-synuclein aggregates; however, it bound soluble α-synuclein at high doses. Biogen later initiated the SPARK Phase II clinical trial (NCT03318523) to further investigate the safety and pharmacokinetics of BIIB054 over two years; however, this trial was discontinued because neither the principal nor ancillary objectives were achieved [118,119].

MEDI1341 is a highly specific, high-affinity mAb (epitope: AA 103-129) that binds monomeric and aggregated α-synuclein, effectively inhibiting its spread. Preclinical studies have confirmed its ability to rapidly penetrate the brain and significantly reduce free α-synuclein levels in cerebrospinal fluid (CSF) and brain interstitial fluid (ISF). MEDI1341 inhibited α-synuclein PFF in SH-SY5Y cell models, while intravenous administration of 100 mg/kg in rats reduced α-synuclein levels by a peak of 81.5% in CSF α-synuclein and 75% in brain ISF. In cynomolgus monkeys, CNS penetration reached 0.2% of serum levels, with up to 88% reduction in CSF α-synuclein. MEDI1341 (TAK-341) is under Phase I clinical trials for safety, pharmacokinetics, and CNS target engagement. These trials involved healthy volunteers and patients with PD and were later modified to include eye health assessments. In November 2022, Takeda took over development, launching a Phase II clinical trial involving 138 patients with MSA, focusing on changes in the Unified MSA Rating Scale and other clinical measures. However, no results have been disclosed at this time [110,120].

AN0805/ABBV-0805 (BAN0805) is a humanized mAb that specifically targets the C-terminus of α-synuclein oligomers and protofibrils. Two different preclinical studies have examined its effects in two mouse models of PD, one expressing human Thy1-A30P and the human α-synuclein^A53T^. The mice received ABBV-0805 at doses from 0.1 to 20 mg/kg over 2-4 months, with treatment effects evaluated through α-synuclein levels, motor function, and survival. ABBV-0805 reduced α-synuclein fibrils, improved motor function, and extended survival, demonstrating its protective effects against α-synuclein toxicity and potential as a PD therapy [110,121].

Lu AF82422 is a human IgG1 mAb that targets all major forms of α-synuclein, including monomeric, oligomeric, and truncated forms. In a single-dose pharmacokinetics (PK) study in cynomolgus monkeys, LuAF82422 demonstrated typical IgG1 mAb properties, including linear PK and a plasma half-life of 10-15 days. Only 0.1%-0.2% of the antibody reached the CSF. In a repeat-dose study, antibody exposure in plasma and CSF increased over time. α-synuclein levels in plasma and CSF showed dose-dependent reductions, but with high variability, possibly due to blood contamination or hemolysis. A significant decrease in free α-synuclein in CSF was observed at doses above 100-600 mg/kg, indicating target engagement. However, the variability in CSF data and the timing of peak drug concentrations (64-88 hours) suggested that higher doses or longer observation periods may be needed for optimal engagement. Modeling estimates suggest that doses of 15-60 mg/kg would effectively bind 85%-95% of oligomeric α-synuclein in CSF, with a safety factor of 10-40 [122].

None of the discussed trials provide clear evidence of these antibodies’ ability to eliminate α-synuclein aggregation through autophagic pathways. There is also a noticeable gap in studies investigating their efficacy against mutated forms of α-synuclein. Therefore, further research is warranted to explore these aspects comprehensively.

The broader implications of immunotherapy for clearing α-synuclein aggregates remain under-researched. Cao et al. reported the development and testing of an antibody (DR5-12D) targeting the Na^+^/K^+^-ATPase (NKA) α1 subunit (ATP1A1), which activates NKA-dependent autophagy [123]. NKA is a transmembrane protein that maintains the ion gradient in CNS cells. Evidence suggests that NKA deprivation can exacerbate neurodegeneration and cause PD [124-126]. NKA has also been reported to modulate autophagy [127,128]. The DR5-12D mAb works by targeting the DR region (^897^DVEDSYGQQWTYEQR^911^) of ATP1A1, stabilizing and activating NKA. This enhances autophagy via the AMPK/mTOR/ULK1 pathway, promoting the degradation of α-synuclein aggregates. DR5-12D improved learning, memory, and motor function in a rodent model of PD induced by α-synuclein PFFs [123].

## Peptide-based therapy for α-synuclein clearance

Small molecules dominate the drug market due to their affordability, bioavailability, and potential for oral administration. However, structural constraints render them inefficient against protein-protein interactions (PPIs). Antibodies and other larger biologics can target external PPIs, but not those that occur intracellularly. Through sophisticated peptide synthesis and purification techniques, short peptides provide a promising compromise; they exhibit high specificity to block PPIs, decreased toxicity, minimal risk of immune response, and economical manufacture [129-131]. In PD studies, peptides have mainly been designed to inhibit α-synuclein aggregation rather than clear it from cells (Table 3). To inhibit α-synuclein aggregation and reduce its toxicity, its N-terminal lipid binding domain was targeted using aS_1-25_ (MDVFMKGLSKAKEGVVAAAEKTKQG) [132], Tat-βsyn-degron (YGRKKRRQRRRRTKSGVYLVGR RRG) [133], and NTR-TP-L and -D (GVLYVGS-Aib) [134]. The NAC domain responsible for amyloid aggregation has been targeted using S37 (GAVVWGVTAVKKKKK), S61 (GAVVWGVTAVGR KKRRQRRRPQ), S62 (GAVVWGVTAVKKGR KKR RQRRRPQ), and S71 (YGRKKRRQRRRAVVT[nme-Gly]VTAVAE) [135]. Recently, Nim et al. targeted its C terminus using PDpep1.3 (DEEIERQLKALG) [136]. K84s (FLVWGCLRGSAIGECVVHGGPPSRH), K102s (FLKRWARSTRWGTASCGGS) [137], and p216 (WFQNRRMKWKK) [138] directly inhibited oligomers, while PSMα3 (MEFVAKLFKFFKDLLGKFLGNN) and LL-37 (LLGDFFRKSKEKIGKEFKRIVQRI KDFLRN LVPRTES) [139] reduced type B oligomer fibrils.

Angiotensin-(1-7) has exhibited neuroprotective effects in PD [140-142]. Continuous infusion (1.1 nmol/0.25 μL/hour) into the substantia nigra decreased α-synuclein aggregation and parkinsonian behaviors in a rotenone-induced rat model. At 100 nM, angiotensin-(1-7) also reversed autophagic dysfunction, reducing α-synuclein accumulation. Western blots revealed increased p62 levels and decreased LC3-II levels in neurons treated with rotenone, suggesting defective autophagy. By regulating LC3-II and p62 levels and dramatically increasing autophagic activity, co-treatment with angiotensin-(1-7) may help to restore autophagic function [143]. Cathepsin L (CTSL) is a downstream lysosomal cysteine protease essential for protein degradation [144]. Its overexpression in human embryonic kidney HEK293F cells expressing enhanced GFP (eGFP)-tagged α-synuclein induced autophagy and promoted clearance of α-synuclein^WT^ [145].

**Table 3:** Peptide-based autophagy inducers.

Peptide	Study model	Dose	Duration of treatment	Therapeutic effects	Refs.
***In vitro* study**					
**Angiotensin-1-7**	Rat midbrain dopaminergic neurons	100 nM	24 hours	Blocking the MAS receptor increased the LC3-II/LC3-I ratio, reduced α-synuclein aggregation in the substantia nigra, improved behavior, induced autophagy, and cleared the aggregated protein.	[143]
**CTSL**	HEK293F/α-synuclein-EGFP cells	Overexpression	-	Induced autophagy via ATG5/LC-3, facilitating the clearance of α-synuclein fibrils from the cell.	[145]
**ATACC peptide**	HEK293T cells	0-100 µM	24 hours	Autolysosome formation, enhanced LC3-II/LC3-I ratio, and degraded α-synuclein aggregates.	[146]
**KYP-2407**	SH-SY5Y cells	10 μM	48 hours	Increased autophagy, normalized CAPN1 and CAPN2 levels, and cleared α-synuclein.	[152]
***In vivo* study**					
**Angiotensin-1-7**	Sprague-Dawley rats	1.1 nmol/0.25 μL/hour, Continuous supranigral infusion	4 weeks	Blocking the MAS receptor increased the LC3-II/LC3-I ratio, reduced α-synuclein aggregation in the substantia nigra, improved behavior, induced autophagy, and cleared the aggregated protein.	[143]
**ATACC peptide**	C57BL/6 mice	50 mg/kg/b.w./day, Intravenous injection	5 days	Autolysosome formation, enhanced LC3-II/LC3-I ratio, and degraded α-synuclein aggregates.	[146]

Abbreviations: α-syn: Alpha synuclein; LC3: Microtubule-associated protein 1A/1B-light chain 3ATG: Autophagy-related protein; b.w: Body weight.

One method for reducing α-synuclein aggregation is targeted protein degradation (TPD). While many proteins are degraded via the ALP, few TPD tools exploit this route. Tong et al. developed four autophagosome-anchoring chimeras (ATACCs). ATACCs promote selective autophagy by employing a bifunctional peptide containing an LC3-interacting region (LIR) to anchor proteins of interest (POIs)-such as α-synuclein-to LC3B on autophagosomes. In both *in vivo* and *in vitro* models, the peptide successfully degraded the aggregated protein via autophagy. An α-synuclein overexpressing HEK293T cell line was used to assess the degrading effects on α-synuclein. α-synuclein was knocked down by all four ATACC peptides in a dose-dependent manner. Peptide P1 exhibited the greatest effectiveness, with a half-maximal degradation concentration (DC_50_) of 46.8 ± 3.7 μM [146].

Prolyl endopeptidase (PREP) is a serine protease that directly interacts with α-synuclein to promote dimerization and aggregation, independent of its enzymatic activity. This interaction is associated with the formation of α-synuclein aggregates observed in patients with PD and dementia with Lewy bodies [147]. PREP inhibitors have been reported to induce autophagy and promote α-synuclein clearance both *in vivo* and *in vitro* [148-151]. Rostami et al. investigated the effect of KYP-2047 on intracellular α-synuclein PFFs in SH-SY5Y cells and human astrocytes. Confirmed by immunostaining and western blots, 10 mM KYP-2047 decreased intracellular α-synuclein deposits in SH-SY5Y cells but not in astrocytes, which lack PREP activity. In addition, KYP-2047 reduced the levels of autophagy-related proteins p62 and light chain 3 beta 2 (LC3B2) in SH-SY5Y cells, indicating increased autophagy. Moreover, the treatment normalized calpain 1 (CAPN1) and 2 (CAPN2) levels, counteracting their increase due to α-synuclein accumulation [152].

Peptide-based therapies offer a promising alternative for inhibiting α-synuclein aggregation and promoting its clearance in PD. TPD- and autophagy-enhancing peptides represent novel strategies to facilitate α-synuclein clearance. Ongoing research continues to explore peptide-based approaches as potential disease-modifying treatments for PD.

## Other autophagy inducers for α-synuclein clearance

Traditional Chinese medicines reportedly act as autophagy inducers to reduce the risk of NDD [153,154]. The ethanol extract of *Artemisia argyi* H. Lév. & Vaniot (60-80 μg/mL) promoted the clearance of α-synuclein aggregates in MPP^+^-induced SH-SY5Y cells in a dose-dependent manner. It also increased levels of TRPML1-a nonselective cation channel located in lysosomal membranes-and enhanced lysosomal activity. Activation of TRPML1 can trigger autophagy, which is significantly increased by *A. argyi* extract [155-157]. Another popularly used traditional Chinese medicine, *C. chinensis* rhizome extract, also triggered autophagy via the TFEB-mediated pathway in SH-SY5Y cells expressing GFP-labeled α-synuclein^A53T^ [158].

Nanoparticles can successfully cross the BBB and are used in PD to deliver active compounds to dopaminergic neuron-rich areas. Various nanoparticles are reported to exhibit different mechanisms for combating PD [150]. Gold nanoclusters were found to have anti-fibrillation properties against α-synuclein aggregation, while melatonin-loaded polymeric nanoparticles reduced α-synuclein fibrillation *in vitro* [159,160]. Gold nanoparticles, such as CNM-Au8, are reported to protect dopaminergic neurons against oxidative stress in MPP^+^- and 6-hydroxydopamine (6-OHDA)-induced lesions [161]. Recently, significant progress has been made in inorganic (metal, quantum dot, and cerium oxide) and organic (polymeric, solid, and liposome) nanoparticles [162]. Quercetin-modified Cu_2_-_X_Se (CSPQ), an enzyme-like nanoparticle, exhibits effective ROS scavenging activity [163] and successfully clears α-synuclein aggregates. In α-synuclein PFF-treated primary neurons isolated from C57BL/6 mice, 4 μM CSPQ nanoparticles significantly reduced α-synuclein aggregation [164].

## New technology for targeted protein degradation (TPD) in PD

Traditional therapies primarily aim to manage symptoms without addressing the underlying cause of protein accumulation. In recent years, TPD has emerged as a promising therapeutic strategy that directly eliminates disease-driving proteins by co-opting a cell’s degradation machinery [165]. Unlike conventional inhibitors that block protein function, TPD approaches-including PROTACs (proteolysis-targeting chimeras), molecular glues, and autophagy-based degraders-aim to selectively and efficiently degrade toxic membrane proteins and aggregated proteins such as α-synuclein. By promoting the clearance of pathogenic proteins, TPD offers the potential for disease modification and neuroprotection in PD, marking a significant advancement in the development of next-generation therapeutics [166].

Autophagosome-tethering compound (ATTEC) technology is an emerging method for degrading POIs. Unlike conventional therapies, ATTECs harness autophagy directly to eliminate specific disease-causing proteins. By linking POIs to the autophagy machinery, ATTECs can potentially target a wide range of proteins, even those previously considered undruggable [167,168]. However, challenges remain in designing target-specific molecules. One study combined an ATTEC with aptamers, highly specific and affinity-purified DNA or RNA oligonucleotides [169], to create targeted protein degraders. Using α-synuclein as a model pathological protein, a biorthogonal click reaction was used to conjugate an α-synuclein-targeting aptamer with the LC3-binding compound 5,7-dihydroxy-4-phenylcoumarin (DP). The resulting aptamer-DP conjugate successfully degraded α-synuclein aggregates through autophagic pathways. This approach demonstrates the potential of aptamer-based ATTECs for designing versatile and specific degraders for various proteins [170].

Recently, an autophagy-targeting chimera (AUTOTAC) has been developed, enabling TPD therapeutics employing p62-mediated autophagy. This compound utilizes a chimeric molecule consisting of a target-binding ligand (TBL) connected to an autophagy-targeting ligand (ATL) that interacts with the ZZ domain of p62 [171]. Anle138b is a chemical compound used as an aggregation inhibitor for α-synuclein and is currently being examined in a Phase 1b clinical trial involving patients with PD (NCT04685265) [172,173]. AUTOTAC uses anle138b as a TBL component to specifically target α-synuclein, while its ATL component binds to p62, promoting its self-oligomerization within its complex. This facilitates the recruitment of aggregates to LC3^+^ autophagic membranes, leading to lysosomal degradation at a concentration of 1 mM in PD model cells (HeLa, COS7, and HEK293T). The degradation process selectively targets detergent-insoluble α-synuclein aggregates without affecting functional, monomeric α-synuclein in SH-SY5Y cells. It also reduced cell-to-cell transmission of α-synuclein aggregates in cultured cells. In PD model mice, oral administration of 10 mg/kg of ATC161 demonstrated therapeutic efficacy by degrading α-synuclein aggregates in the brain, improving glial immune responses, and slowing the progression of motor deficits [174].

## Limitations and future perspectives

Proteins targeted by CMA typically contain a recognition motif that is identified by the HSC70 chaperone, which guides them to the lysosomal membrane. α-synuclein possesses such a motif and is normally degraded via CMA. Disease-associated mutant forms of α-synuclein, such as α-synuclein^A53T^, are still capable of binding to the LAMP2A receptor but exhibit severely impaired translocation into the lysosomal lumen. As a result, these mutant proteins accumulate at the lysosomal surface and can interfere with normal CMA functioning, contributing to protein aggregation and cellular stress in neurodegenerative conditions [175,176]. This results in the activation of macroautophagy as an alternative degradation pathway. Unfortunately, it has been demonstrated that α-synuclein^A53T^ and α-synuclein^E46K^ aggregates are not degraded via autophagy, even when a lysosomal cysteine protease, CTSL, is overexpressed [145,177]. Therefore, thoroughly investigating autophagy activation for mutant α-synuclein degradation is crucial. Additionally, few studies have examined the clearance of other mutant α-synuclein aggregates. For instance, no studies have explored the clearance of the aggregated form of α-synuclein^A30P^, another aggregate-prone mutant, with reports only published on its monomer form [178,179].

Studying the long-term effects of hyperactive autophagy on cell survival is necessary, as excessive autophagy has been associated with various mechanisms of cell death. This has led to the use of the term “autophagic cell death” to describe cell death occurring alongside autophagy [180,181]. Overactive autophagy can be pathogenic [182], and chronic activation of the process can lead to neurodegeneration and neuronal death. Zhang et al. reported that chronic corticosterone (CORT) treatment of C57BL/6J mice hyperactivated autophagy by increasing autophagy-related 5 (*ATG5*) expression. This led to excess lysosomal degradation of brain-derived neurotrophic factor (BDNF) in dopaminergic neurons and subsequently impaired neurogenesis [183]. Therefore, research is needed to determine the point at which autophagy regulation is optimized to maintain neuronal function.

Inhibiting α-synuclein aggregation is the most common approach to preventing PD progression. However, these aggregates are already present in the brains of patients by the time symptoms manifest. Aggregation inhibitors may prevent new aggregates forming but cannot remove those already present. Enhancing autophagic clearance can actively degrade pre-existing toxic aggregates, potentially reversing or mitigating disease progression. In addition, autophagic pathways can target a range of α-synuclein forms, including smaller toxic intermediates and larger aggregates. Associated treatments would be applicable even in advanced stages, targeting existing aggregates regardless of disease progression. Therefore, further *in vivo/in vitro* studies exploring how α-synuclein degradation can be enhanced are also important. While strategies to promote α-synuclein clearance via autophagy activation are proposed as treatments, there is currently limited evidence regarding their clinical applicability and side effects.

## Conclusion

Recent advances in understanding the clearance of α-synuclein aggregates via autophagy have highlighted its potential as a therapeutic strategy for NDDs such as PD and MSA. Autophagy-enhancing strategies hold strong therapeutic potential for the clearance of pathological α-synuclein in PD. Emerging approaches-such as ATACC, ATTEC, AUTOTAC, and CMA—offer novel and selective means to degrade aggregated or misfolded α-synuclein, including forms that are otherwise resistant to conventional clearance mechanisms. However, challenges remain, including the difficulty of identifying specific toxic α-synuclein species, the diverse nature of α-synuclein aggregation between patients, and the lack of reliable biomarkers for monitoring treatment efficacy. Despite growing interest in immunotherapeutic approaches for PD, there remains a significant gap in studies specifically targeting the elimination of mutant forms of α-synuclein. Likewise, the exploration of autophagy-based mechanisms for the selective degradation of these pathogenic variants is still in its infancy. Although most of the compounds discussed above have demonstrated adequate safety and tolerability profiles, only a limited number have shown the ability to effectively cross the BBB, a critical requirement for therapeutic efficacy in NDDs. Future research should focus on elucidating the molecular mechanisms regulating autophagy in α-synuclein-related pathologies and optimizing therapeutic interventions that leverage this pathway to enhance the clearance of pathological aggregates.

## References

[1] Calabresi P, Mechelli A, Natale G, Volpicelli-Daley L, Di Lazzaro G, Ghiglieri V (2023). Alpha-synuclein in Parkinson’s disease and other synucleinopathies: from overt neurodegeneration back to early synaptic dysfunction. Cell Death Dis, 14:176. 10.1038/s41419-023-05672-9 PMC9977911 36859484

[2] Baggett D, Olson A, Parmar MS (2024). Novel approaches targeting α-Synuclein for Parkinson’s Disease: Current progress and future directions for the disease-modifying therapies. Brain Disord, 16:100163.

[3] Ebrahimi-Fakhari D, Cantuti-Castelvetri I, Fan Z, Rockenstein E, Masliah E, Hyman BT, et al. (2011). Distinct Roles In Vivo for the Ubiquitin-Proteasome System and the Autophagy-Lysosomal Pathway in the Degradation of α-Synuclein. J Neurosci, 31:14508-14520. 10.1523/JNEUROSCI.1560-11.2011 PMC3587176 21994367

[4] Grosso Jasutkar H, Oh SE, Mouradian MM (2022). Therapeutics in the Pipeline Targeting α-Synuclein for Parkinson’s Disease. Pharmacol Rev, 74:207-237. 10.1124/pharmrev.120.000133 PMC11034868 35017177

[5] Emmanouilidou E, Stefanis L, Vekrellis K (2010). Cell-produced α-synuclein oligomers are targeted to, and impair, the 26S proteasome. Neurobiol Aging, 31:953-968. 10.1016/j.neurobiolaging.2008.07.008 18715677

[6] Hinault M-P, Cuendet AFH, Mattoo RUH, Mensi M, Dietler G, Lashuel HA, et al. (2010). Stable α-Synuclein Oligomers Strongly Inhibit Chaperone Activity of the Hsp70 System by Weak Interactions with J-domain Co-chaperones. J Biol Chem, 285:38173-38182. 10.1074/jbc.M110.127753 PMC2992251 20847048

[7] Yoo JM, Lin Y, Heo Y, Lee Y-H (2022). Polymorphism in alpha-synuclein oligomers and its implications in toxicity under disease conditions. Front Mol Biosci, 9:959425. 10.3389/fmolb.2022.959425 PMC9412080 36032665

[8] Mehra S, Gadhe L, Bera R, Sawner AS, Maji SK (2021). Structural and Functional Insights into α-Synuclein Fibril Polymorphism. Biomolecules, 11:1419. 10.3390/biom11101419 PMC8533119 34680054

[9] Gao J, Perera G, Bhadbhade M, Halliday GM, Dzamko N (2019). Autophagy activation promotes clearance of α-synuclein inclusions in fibril-seeded human neural cells. J Biol Chem, 294:14241-14256. 10.1074/jbc.RA119.008733 PMC6768637 31375560

[10] Boland B, Yu WH, Corti O, Mollereau B, Henriques A, Bezard E, et al. (2018). Promoting the clearance of neurotoxic proteins in neurodegenerative disorders of ageing. Nat Rev Drug Discov, 17:660-688. 10.1038/nrd.2018.109 PMC6456907 30116051

[11] Dinter E, Saridaki T, Nippold M, Plum S, Diederichs L, Komnig D, et al. (2016). Rab7 induces clearance of α-synuclein aggregates. J Neurochem, 138:758-774. 10.1111/jnc.13712 27333324

[12] Szegö EM, Van den Haute C, Höfs L, Baekelandt V, Van der Perren A, Falkenburger BH (2022). Rab7 reduces α-synuclein toxicity in rats and primary neurons. Exp Neurol, 347:113900. 10.1016/j.expneurol.2021.113900 34695425

[13] Aman Y, Schmauck-Medina T, Hansen M, Morimoto RI, Simon AK, Bjedov I, et al. (2021). Autophagy in healthy aging and disease. Nat Aging, 1:634-650. 10.1038/s43587-021-00098-4 PMC8659158 34901876

[14] Winslow AR, Chen C-W, Corrochano S, Acevedo-Arozena A, Gordon DE, Peden AA, et al. (2010). α-Synuclein impairs macroautophagy: implications for Parkinson’s disease. J Cell Biol, 190:1023-1037. 10.1083/jcb.201003122 PMC3101586 20855506

[15] Ahmed I, Liang Y, Schools S, Dawson VL, Dawson TM, Savitt JM (2012). Development and Characterization of a New Parkinson’s Disease Model Resulting from Impaired Autophagy. J Neurosci, 32:16503-16509. 10.1523/JNEUROSCI.0209-12.2012 PMC3508432 23152632

[16] Sato S, Uchihara T, Fukuda T, Noda S, Kondo H, Saiki S, et al. (2018). Loss of autophagy in dopaminergic neurons causes Lewy pathology and motor dysfunction in aged mice. Sci Rep, 8:2813. 10.1038/s41598-018-21325-w PMC5809579 29434298

[17] Madureira M, Connor-Robson N, Wade-Martins R (2020). “LRRK2: Autophagy and Lysosomal Activity.” Front Neurosci, 14:498. 10.3389/fnins.2020.00498 PMC7262160 32523507

[18] Pang SY-Y, Lo RCN, Ho PW-L, Liu H-F, Chang EES, Leung C-T, et al. (2022). LRRK2, GBA and their interaction in the regulation of autophagy: implications on therapeutics in Parkinson’s disease. Transl Neurodegener, 11:5. 10.1186/s40035-022-00281-6 PMC8805403 35101134

[19] Smith L, Schapira AH V. (2022). GBA Variants and Parkinson Disease: Mechanisms and Treatments. Cells, 11:1261. 10.3390/cells11081261 PMC9029385 35455941

[20] Höglinger G, Schulte C, Jost WH, Storch A, Woitalla D, Krüger R, et al. (2022). GBA-associated PD: chances and obstacles for targeted treatment strategies. J Neural Transm, 129:1219-1233. 10.1007/s00702-022-02511-7 PMC9463270 35639160

[21] Matoba K, Kotani T, Tsutsumi A, Tsuji T, Mori T, Noshiro D, et al. (2020). Atg9 is a lipid scramblase that mediates autophagosomal membrane expansion. Nat Struct Mol Biol, 27:1185-1193. 10.1038/s41594-020-00518-w 33106658

[22] Tang Q, Gao P, Arzberger T, Höllerhage M, Herms J, Höglinger G, et al. (2021). Alpha-Synuclein defects autophagy by impairing SNAP29-mediated autophagosome-lysosome fusion. Cell Death Dis, 12:854. 10.1038/s41419-021-04138-0 PMC8448865 34535638

[23] Colonna M, Butovsky O (2017). Microglia Function in the Central Nervous System During Health and Neurodegeneration. Annu Rev Immunol, 35:441-468. 10.1146/annurev-immunol-051116-052358 PMC8167938 28226226

[24] Tu H, Yuan B, Hou X, Zhang X, Pei C, Ma Y, et al. (2021). α-synuclein suppresses microglial autophagy and promotes neurodegeneration in a mouse model of Parkinson’s disease. Aging Cell, 12:e13522. 10.1111/acel.13522 PMC8672776 34811872

[25] Flagmeier P, Meisl G, Vendruscolo M, Knowles TPJ, Dobson CM, Buell AK, et al. (2016). Mutations associated with familial Parkinson’s disease alter the initiation and amplification steps of α-synuclein aggregation. Proc Natl Acad Sci, 113:10328-10333. 10.1073/pnas.1604645113 PMC5027465 27573854

[26] Sen S, Dey A, Maulik U (2021). Studying the effect of alpha-synuclein and Parkinson’s disease linked mutants on inter pathway connectivities. Sci Rep, 11:16365. 10.1038/s41598-021-95889-5 PMC8358055 34381149

[27] Chauhan S, Goodwin JG, Chauhan S, Manyam G, Wang J, Kamat AM, et al. (2013). ZKSCAN3 Is a Master Transcriptional Repressor of Autophagy. Mol Cell, 50:16-28. 10.1016/j.molcel.2013.01.024 PMC3628091 23434374

[28] Lei Z, Cao G, Wei G (2019). A30P mutant α-synuclein impairs autophagic flux by inactivating JNK signaling to enhance ZKSCAN3 activity in midbrain dopaminergic neurons. Cell Death Dis, 10:133. 10.1038/s41419-019-1364-0 PMC6372582 30755581

[29] Rodríguez-Navarro JA, Rodríguez L, Casarejos MJ, Solano RM, Gómez A, Perucho J, et al. (2010). Trehalose ameliorates dopaminergic and tau pathology in parkin deleted/tau overexpressing mice through autophagy activation. Neurobiol Dis, 39:423-438. 10.1016/j.nbd.2010.05.014 20546895

[30] Lin C-H, Wu Y-R, Yang J-M, Chen W-L, Chao C-Y, Chen I-C, et al. (2016). Novel Lactulose and Melibiose Targeting Autophagy to Reduce PolyQ Aggregation in Cell Models of Spinocerebellar Ataxia 3. CNS Neurol Disord Drug Targets, 15:351-359. 10.2174/1871527314666150821101522 26295831

[31] Lee G-C, Lin C-H, Tao Y-C, Yang J-M, Hsu K-C, Huang Y-J, et al. (2015). The potential of lactulose and melibiose, two novel trehalase-indigestible and autophagy-inducing disaccharides, for polyQ-mediated neurodegenerative disease treatment. Neurotoxicology, 48:120-130. 10.1016/j.neuro.2015.03.009 25800379

[32] Chen CM, Lin C-H, Wu Y-R, Yen C-Y, Huang Y-T, Lin J-L, et al. (2020). Lactulose and Melibiose Inhibit α-Synuclein Aggregation and Up-Regulate Autophagy to Reduce Neuronal Vulnerability. Cells, 9:1230. 10.3390/cells9051230 PMC7290909 32429337

[33] Tang D, Kang R, Livesey KM, Cheh C-W, Farkas A, Loughran P, et al. (2010). Endogenous HMGB1 regulates autophagy. J Cell Biol, 190:881-892. 10.1083/jcb.200911078 PMC2935581 20819940

[34] Lu J, He L, Behrends C, Araki M, Araki K, Jun Wang Q, et al. (2014). NRBF2 regulates autophagy and prevents liver injury by modulating Atg14L-linked phosphatidylinositol-3 kinase III activity. Nat Commun, 5:3920. 10.1038/ncomms4920 PMC4376476 24849286

[35] Kang R, Zeh HJ, Lotze MT, Tang D (2011). The Beclin 1 network regulates autophagy and apoptosis. Cell Death Differ, 18:571-580. 10.1038/cdd.2010.191 PMC3131912 21311563

[36] Song J-X, Lu J-H, Liu L-F, Chen L-L, Durairajan SSK, Yue Z, et al. (2014). HMGB1 is involved in autophagy inhibition caused by SNCA/α-synuclein overexpression. Autophagy, 10:144-154. 10.4161/auto.26751 PMC4389868 24178442

[37] Zhu Q, Song J, Chen J-Y, Yuan Z, Liu L, Xie L-M, et al. (2023). Corynoxine B targets at HMGB1/2 to enhance autophagy for α-synuclein clearance in fly and rodent models of Parkinson’s disease. Acta Pharm Sin B, 13:2701-2714. 10.1016/j.apsb.2023.03.011 PMC10326294 37425041

[38] Miao Q, Chai Z, Song L-J, Wang Q, Song G-B, Wang J, et al. (2022). The neuroprotective effects and transdifferentiation of astrocytes into dopaminergic neurons of Ginkgolide K on Parkinson’ disease mice. J Neuroimmunol, 364:577806. 10.1016/j.jneuroim.2022.577806 35121334

[39] Miao Q, Li X-H, Wang J, Wang Q, Chai Z, Li Y-Q, et al. (2021). The neuroprotective effects of Ginkgolide K on MPTP mice. J Neurol Sci, 429:119478.

[40] Zhang Y, Miao J-M (2018). Ginkgolide K promotes astrocyte proliferation and migration after oxygen-glucose deprivation via inducing protective autophagy through the AMPK/mTOR/ULK1 signaling pathway. Eur J Pharmacol, 832:96-103. 10.1016/j.ejphar.2018.05.029 29787772

[41] Yu W, Chen S, Cao L, Tang J, Xiao W, Xiao B (2018). Ginkgolide K promotes the clearance of A53T mutation alpha-synuclein in SH-SY5Y cells. Cell Biol Toxicol, 34:291-303. 10.1007/s10565-017-9419-4 29214369

[42] Iwenofu OH, Lackman RD, Staddon AP, Goodwin DG, Haupt HM, Brooks JSJ (2008). Phospho-S6 ribosomal protein: a potential new predictive sarcoma marker for targeted mTOR therapy. Modern Pathology, 21:231-237. 10.1038/modpathol.3800995 18157089

[43] Leri M, Vasarri M, Palazzi L, Barletta E, Nielsen E, Bucciantini M, et al. (2020). Maysin plays a protective role against α-Synuclein oligomers cytotoxicity by triggering autophagy activation. Food Chem Toxicol, 144:111626. 10.1016/j.fct.2020.111626 32738375

[44] Wang X, Hu W, Qu L, Wang J, Wu A, Lo HH, et al. (2023). Tricin promoted ATG-7 dependent autophagic degradation of α-synuclein and dopamine release for improving cognitive and motor deficits in Parkinson’s disease. Pharmacol Res, 196:106874. 10.1016/j.phrs.2023.106874 37586619

[45] Ciaramella A, Salani F, Bizzoni F, Pontieri FE, Stefani A, Pierantozzi M, et al. (2013). Blood Dendritic Cell Frequency Declines in Idiopathic Parkinson’s Disease and Is Associated with Motor Symptom Severity. PLoS One, 8:e65352. 10.1371/journal.pone.0065352 PMC3679103 23776473

[46] Harms AS, Thome AD, Yan Z, Schonhoff AM, Williams GP, Li X, et al. (2018). Peripheral monocyte entry is required for alpha-Synuclein induced inflammation and Neurodegeneration in a model of Parkinson disease. Exp Neurol, 300:179-187. 10.1016/j.expneurol.2017.11.010 PMC5759972 29155051

[47] Ng L, Wang X, Yang C, Su C, Li M, Cheung AKL (2022). Corrigendum: Celastrol Downmodulates Alpha-Synuclein-Specific T Cell Responses by Mediating Antigen Trafficking in Dendritic Cells. Front Immunol, 13:907993. 10.3389/fimmu.2022.907993 PMC9164218 35669764

[48] Lin C-H, Wu Y-R, Chao C-Y, Chang K-H, Chen C-M, Chen W-L, et al. (2024). Protective Effects of *Coptis chinensis* Rhizome Extract and Its Constituents (Berberine, Coptisine, and Palmatine) against α-Synuclein Neurotoxicity in Dopaminergic SH-SY5Y Cells. Biol Pharm Bull, 47:b23-00758. 10.1248/bpb.b23-00758 38599826

[49] Guo Y-L, Duan W-J, Lu D-H, Ma X-H, Li X-X, Li Z, et al. (2021). Autophagy-dependent removal of α-synuclein: a novel mechanism of GM1 ganglioside neuroprotection against Parkinson’s disease. Acta Pharmacol Sin, 42:518-528. 10.1038/s41401-020-0454-y PMC8115090 32724177

[50] Kimura S, Noda T, Yoshimori T (2008). Dynein-dependent Movement of Autophagosomes Mediates Efficient Encounters with Lysosomes. Cell Struct Funct, 33:109-122. 10.1247/csf.08005 18388399

[51] Korolchuk VI, Saiki S, Lichtenberg M, Siddiqi FH, Roberts EA, Imarisio S, et al. (2011). Lysosomal positioning coordinates cellular nutrient responses. Nat Cell Biol, 13:453-460. 10.1038/ncb2204 PMC3071334 21394080

[52] Olanow CW, Perl DP, DeMartino GN, McNaught KSP (2004). Lewy-body formation is an aggresome-related process: a hypothesis. Lancet Neurol, 3:496-503. 10.1016/S1474-4422(04)00827-0 15261611

[53] Date Y, Sasazawa Y, Kitagawa M, Gejima K, Suzuki A, Saya H, et al. (2024). Novel autophagy inducers by accelerating lysosomal clustering against Parkinson’s disease. Elife. 13:e98649. 10.7554/eLife.98649 PMC11221835 38899618

[54] Sasazawa Y, Date Y, Hattori N, Saiki S (2024). Clustering lysosomes around the MTOC: a promising strategy for SNCA/alpha-synuclein breakdown leading to parkinson disease treatment. Autophagy, 20:2839-2840. 10.1080/15548627.2024.2413295 PMC11587859 39401987

[55] Harrison IF, Dexter DT (2013). Epigenetic targeting of histone deacetylase: Therapeutic potential in Parkinson’s disease? Pharmacol Ther, 140:34-52. 10.1016/j.pharmthera.2013.05.010 23711791

[56] Füllgrabe J, Heldring N, Hermanson O, Joseph B (2014). Cracking the survival code. Autophagy, 10:556-561. 10.4161/auto.27280 PMC4091144 24429873

[57] Sando R, Gounko N, Pieraut S, Liao L, Yates J, Maximov A (2012). HDAC4 Governs a Transcriptional Program Essential for Synaptic Plasticity and Memory. Cell, 151:821-834. 10.1016/j.cell.2012.09.037 PMC3496186 23141539

[58] Wu Q, Yang X, Zhang L, Zhang Y, Feng L (2017). Nuclear Accumulation of Histone Deacetylase 4 (HDAC4) Exerts Neurotoxicity in Models of Parkinson’s Disease. Mol Neurobiol, 54:6970-6983. 10.1007/s12035-016-0199-2 27785754

[59] Wang L, Liu L, Han C, Jiang H, Ma K, Guo S, et al. (2023). Histone Deacetylase 4 Inhibition Reduces Rotenone-Induced Alpha-Synuclein Accumulation via Autophagy in SH-SY5Y Cells. Brain Sci, 13:670. 10.3390/brainsci13040670 PMC10136981 37190635

[60] Suresh SN, Manjithaya R (2019). A small molecule autophagy inducer exerts cytoprotection against α-synuclein toxicity. Eur J Pharmacol, 862:172635. 10.1016/j.ejphar.2019.172635 31491404

[61] Kumar A V., Mills J, Lapierre LR (2022). Selective Autophagy Receptor p62/SQSTM1, a Pivotal Player in Stress and Aging. Front Cell Dev Biol, 10:793328. 10.3389/fcell.2022.793328 PMC8883344 35237597

[62] Cheng X-T, Xie Y-X, Zhou B, Huang N, Farfel-Becker T, Sheng Z-H (2018). Revisiting LAMP1 as a marker for degradative autophagy-lysosomal organelles in the nervous system. Autophagy, 14:1472-1474. 10.1080/15548627.2018.1482147 PMC6103665 29940787

[63] Hu Q, Xu L, Liu X, Wang Y, Yuan J (2024). Adenosine A2A receptor antagonist KW6002 protects against A53T mutant alpha-synuclein-induced brain damage and neuronal apoptosis in Parkinson’s disease mice by restoring autophagic flux. Neurosci Lett, 826:137610. 10.1016/j.neulet.2023.137610 38157926

[64] Xu J, Ao Y-L, Huang C, Song X, Zhang G, Cui W, et al. (2022). Harmol promotes α-synuclein degradation and improves motor impairment in Parkinson’s models via regulating autophagy-lysosome pathway. NPJ Parkinsons Dis, 8:100. 10.1038/s41531-022-00361-4 PMC9357076 35933473

[65] Wang X, Cao G, Ding D, Li F, Zhao X, Wang J, et al. (2022). Ferruginol prevents degeneration of dopaminergic neurons by enhancing clearance of α-synuclein in neuronal cells. Fitoterapia, 156:105066. 10.1016/j.fitote.2021.105066 34678438

[66] Sawin ER, Ranganathan R, Horvitz HR (2000). C. elegans Locomotory Rate Is Modulated by the Environment through a Dopaminergic Pathway and by Experience through a Serotonergic Pathway. Neuron, 26:619-631. 10.1016/s0896-6273(00)81199-x 10896158

[67] Chalorak P, Sornkaew N, Manohong P, Niamnont N, Malaiwong N, Limboonreung T, et al. (2021). Diterpene glycosides from Holothuria scabra exert the α-synuclein degradation and neuroprotection against α-synuclein-Mediated neurodegeneration in C. elegans model. J Ethnopharmacol, 279:114347. 10.1016/j.jep.2021.114347 PMC8381228 34147616

[68] Antonioli L, Blandizzi C, Fornai M, Pacher P, Lee HT, Haskó G (2019). P2X4 receptors, immunity, and sepsis. Curr Opin Pharmacol, 47:65-74. 10.1016/j.coph.2019.02.011 PMC6708460 30921560

[69] Murrell-Lagnado RD, Frick M (2019). P2X4 and lysosome fusion. Curr Opin Pharmacol, 47:126-132. 10.1016/j.coph.2019.03.002 31039505

[70] Huang P, Zou Y, Zhong XZ, Cao Q, Zhao K, Zhu MX, et al. (2014). P2X4 Forms Functional ATP-activated Cation Channels on Lysosomal Membranes Regulated by Luminal pH. J Biol Chem, 289:17658-17667. 10.1074/jbc.M114.552158 PMC4067200 24817123

[71] Zhong XZ, Yang Y, Sun X, Dong X-P (2017). Methods for monitoring Ca 2+ and ion channels in the lysosome. Cell Calcium, 64:20-28. 10.1016/j.ceca.2016.12.001 27986285

[72] Li R, Lu Y, Zhang Q, Liu W, Yang R, Jiao J, et al. (2022). Piperine promotes autophagy flux by P2RX4 activation in SNCA /α-synuclein-induced Parkinson disease model. Autophagy, 18:559-575. 10.1080/15548627.2021.1937897 PMC9037522 34092198

[73] Wu J-Z, Ardah M, Haikal C, Svanbergsson A, Diepenbroek M, Vaikath NN, et al. (2019). Dihydromyricetin and Salvianolic acid B inhibit alpha-synuclein aggregation and enhance chaperone-mediated autophagy. Transl Neurodegener, 8:18. 10.1186/s40035-019-0159-7 PMC6570948 31223479

[74] Novellino F, Saccà V, Donato A, Zaffino P, Spadea MF, Vismara M, et al. (2020). Innate Immunity: A Common Denominator between Neurodegenerative and Neuropsychiatric Diseases. Int J Mol Sci, 21:1115. 10.3390/ijms21031115 PMC7036760 32046139

[75] Ganguly U, Singh S, Chakrabarti S, Saini AK, Saini R V. (2022). Immunotherapeutic interventions in Parkinson’s disease: Focus on α-Synuclein. Adv Protein Chem Struct Biol, 129:381-433. 10.1016/bs.apcsb.2021.11.010 35305723

[76] Subramaniam SR, Federoff HJ (2017). Targeting Microglial Activation States as a Therapeutic Avenue in Parkinson’s Disease. Front Aging Neurosci, 9:176. 10.3389/fnagi.2017.00176 PMC5463358 28642697

[77] Saitgareeva AR, Bulygin K V., Gareev IF, Beylerli OA, Akhmadeeva LR (2020). The role of microglia in the development of neurodegeneration. Neurol Sci, 41:3609-3615. 10.1007/s10072-020-04468-5 32458252

[78] Lv Q-K, Tao K-X, Wang X-B, Yao X-Y, Pang M-Z, Liu J-Y, et al. (2023). Role of α-synuclein in microglia: autophagy and phagocytosis balance neuroinflammation in Parkinson’s disease. Inflamm Res, 72:443-462. 10.1007/s00011-022-01676-x 36598534

[79] Lin D, Zhang H, Zhang J, Huang K, Chen Y, Jing X, et al. (2023). α-Synuclein Induces Neuroinflammation Injury through the IL6ST-AS/STAT3/HIF-1α Axis. Int J Mol Sci, 24:1436. 10.3390/ijms24021436 PMC9861378 36674945

[80] Li Y, Xia Y, Yin S, Wan F, Hu J, Kou L, et al. (2021). Targeting Microglial α-Synuclein/TLRs/NF-kappaB/NLRP3 Inflammasome Axis in Parkinson’s Disease. Front Immunol, 12:719807. 10.3389/fimmu.2021.719807 PMC8531525 34691027

[81] Fellner L, Irschick R, Schanda K, Reindl M, Klimaschewski L, Poewe W, et al. (2013). Toll-like receptor 4 is required for α-synuclein dependent activation of microglia and astroglia. Glia. 61:349-60. 10.1002/glia.22437 PMC3568908 23108585

[82] Xiang H. (2025). The interplay between α-synuclein aggregation and necroptosis in Parkinson’s disease: a spatiotemporal perspective. Front Neurosci. 19:1567445. 10.3389/fnins.2025.1567445 PMC12011736 40264913

[83] Chavarría C, Ivagnes R, Souza JM (2022). Extracellular Alpha-Synuclein: Mechanisms for Glial Cell Internalization and Activation. Biomolecules, 12:655. 10.3390/biom12050655 PMC9138387 35625583

[84] Peña-Martinez C, Rickman AD, Heckmann BL (2022). Beyond autophagy: LC3-associated phagocytosis and endocytosis. Sci Adv, 8:eabn1702. 10.1126/sciadv.abn1702 PMC9604515 36288309

[85] Janda E, Boi L, Carta AR (2018). Microglial Phagocytosis and Its Regulation: A Therapeutic Target in Parkinson’s Disease? Front Mol Neurosci, 11:144. 10.3389/fnmol.2018.00144 PMC5934476 29755317

[86] Choi I, Zhang Y, Seegobin SP, Pruvost M, Wang Q, Purtell K, et al. (2020). Microglia clear neuron-released α-synuclein via selective autophagy and prevent neurodegeneration. Nat Commun, 11:1386. 10.1038/s41467-020-15119-w PMC7069981 32170061

[87] Choi I, Seegobin SP, Liang D, Yue Z (2020). Synucleinphagy: a microglial “community cleanup program” for neuroprotection. Autophagy, 16:1718-1720. 10.1080/15548627.2020.1774149 PMC8386613 32453651

[88] Rocha SM, Kirkley KS, Chatterjee D, Aboellail TA, Smeyne RJ, Tjalkens RB (2023). Microglia-specific knock-out of NF-κB/IKK2 increases the accumulation of misfolded α-synuclein through the inhibition of p62/ sequestosome-1-dependent autophagy in the rotenone model of Parkinson’s disease. Glia, 71:2154-2179. 10.1002/glia.24385 PMC10330367 37199240

[89] Bantle CM, Rocha SM, French CT, Phillips AT, Tran K, Olson KE, et al. (2021). Astrocyte inflammatory signaling mediates α-synuclein aggregation and dopaminergic neuronal loss following viral encephalitis. Exp Neurol, 346:113845. 10.1016/j.expneurol.2021.113845 PMC9535678 34454938

[90] Huang J, Ding J, Wang X, Gu C, He Y, Li Y, et al. (2022). Transfer of neuron-derived α-synuclein to astrocytes induces neuroinflammation and blood-brain barrier damage after methamphetamine exposure: Involving the regulation of nuclear receptor-associated protein 1. Brain Behav Immun, 106:247-261. 10.1016/j.bbi.2022.09.002 36089218

[91] Chou T-W, Chang NP, Krishnagiri M, Patel AP, Lindman M, Angel JP, et al. (2021). Fibrillar α-synuclein induces neurotoxic astrocyte activation via RIP kinase signaling and NF-κB. Cell Death Dis, 12:756. 10.1038/s41419-021-04049-0 PMC8325686 34333522

[92] Weiss F, Hughes L, Fu Y, Bardy C, Halliday GM, Dzamko N (2024). Astrocytes contribute to toll-like receptor 2-mediated neurodegeneration and alpha-synuclein pathology in a human midbrain Parkinson’s model. Transl Neurodegener, 13:62. 10.1186/s40035-024-00448-3 PMC11648304 39681872

[93] Zella MAS, Metzdorf J, Ostendorf F, Maass F, Muhlack S, Gold R, et al. (2019). Novel Immunotherapeutic Approaches to Target Alpha-Synuclein and Related Neuroinflammation in Parkinson’s Disease. Cells, 8:105. 10.3390/cells8020105 PMC6406239 30708997

[94] Giusti V, Kaur G, Giusto E, Civiero L (2024). Brain clearance of protein aggregates: a close-up on astrocytes. Mol Neurodegener, 19:5. 10.1186/s13024-024-00703-1 PMC10790381 38229094

[95] Tsunemi T, Ishiguro Y, Yoroisaka A, Valdez C, Miyamoto K, Ishikawa K, et al. (2020). Astrocytes Protect Human Dopaminergic Neurons from α-Synuclein Accumulation and Propagation. J Neurosci, 40:8618-8628. 10.1523/JNEUROSCI.0954-20.2020 PMC7643299 33046546

[96] Hua J, Yin N, Xu S, Chen Q, Tao T, Zhang J, et al. (2019). Enhancing the Astrocytic Clearance of Extracellular α-Synuclein Aggregates by Ginkgolides Attenuates Neural Cell Injury. Cell Mol Neurobiol, 39:1017-1028. 10.1007/s10571-019-00696-2 PMC11457828 31165943

[97] Yang Y, Song J-J, Choi YR, Kim S, Seok M-J, Wulansari N, et al. (2022). Therapeutic functions of astrocytes to treat α-synuclein pathology in Parkinson’s disease. Proc Natl Acad Sci, 119:e2110746119. 10.1073/pnas.2110746119 PMC9304026 35858361

[98] Baxter D (2007). Active and passive immunity, vaccine types, excipients and licensing. Occup Med (Chic Ill), 57:552-556. 10.1093/occmed/kqm110 18045976

[99] Wang Z, Gao G, Duan C, Yang H (2019). Progress of immunotherapy of anti-α-synuclein in Parkinson’s disease. Biomed Pharmacother, 115:108843. 10.1016/j.biopha.2019.108843 31055236

[100] Mandler M, Valera E, Rockenstein E, Mante M, Weninger H, Patrick C, et al. (2015). Active immunization against alpha-synuclein ameliorates the degenerative pathology and prevents demyelination in a model of multiple system atrophy. Mol Neurodegener, 10:10. 10.1186/s13024-015-0008-9 PMC4411775 25886309

[101] Masliah E, Rockenstein E, Adame A, Alford M, Crews L, Hashimoto M, et al. (2005). Effects of α-Synuclein Immunization in a Mouse Model of Parkinson’s Disease. Neuron, 46:857-868. 10.1016/j.neuron.2005.05.010 15953415

[102] Mandler M, Valera E, Rockenstein E, Weninger H, Patrick C, Adame A, et al. (2014). Next-generation active immunization approach for synucleinopathies: implications for Parkinson’s disease clinical trials. Acta Neuropathol, 127:861-879. 10.1007/s00401-014-1256-4 PMC4034750 24525765

[103] Chatterjee D, Kordower JH (2019). Immunotherapy in Parkinson’s disease: Current status and future directions. Neurobiol Dis, 132:104587. 10.1016/j.nbd.2019.104587 31454546

[104] Su R, Zhou T (2021). Alpha-Synuclein Induced Immune Cells Activation and Associated Therapy in Parkinson’s Disease. Front Aging Neurosci, 13:769506. 10.3389/fnagi.2021.769506 PMC8602361 34803660

[105] Lemos M, Venezia S, Refolo V, Heras-Garvin A, Schmidhuber S, Giese A, et al. (2020). Targeting α-synuclein by PD03 AFFITOPE® and Anle138b rescues neurodegenerative pathology in a model of multiple system atrophy: clinical relevance. Transl Neurodegener, 9:38. 10.1186/s40035-020-00217-y PMC7513530 32972456

[106] Meissner WG, Traon AP, Foubert-Samier A, Galabova G, Galitzky M, Kutzelnigg A, et al. (2020). A Phase 1 Randomized Trial of Specific Active α-Synuclein Immunotherapies PD01A and PD03Ain Multiple System Atrophy. Mov Disord, 35:1957-1965. 10.1002/mds.28218 PMC7754431 32882100

[107] Poewe W, Volc D, Seppi K, Medori R, Lührs P, Kutzelnigg A, et al. (2021). Safety and Tolerability of Active Immunotherapy Targeting α-Synuclein with PD03A in Patients with Early Parkinson’s Disease: A Randomized, Placebo-Controlled, Phase 1 Study. J Parkinsons Dis, 11:1079-1089. 10.3233/JPD-212594 PMC8461711 34092654

[108] Nimmo JT, Smith H, Wang CY, Teeling JL, Nicoll JAR, Verma A, et al. (2022). Immunisation with UB-312 in the Thy1SNCA mouse prevents motor performance deficits and oligomeric α-synuclein accumulation in the brain and gut. Acta Neuropathol, 143:55-73. 10.1007/s00401-021-02381-5 PMC8732825 34741635

[109] Yu HJ, Thijssen E, van Brummelen E, van der Plas JL, Radanovic I, Moerland M, et al. (2022). A Randomized First-in-Human Study With UB-312, a UBITh® α-Synuclein Peptide Vaccine. Mov Disord, 37:1416-1424. 10.1002/mds.29016 PMC9545051 35426173

[110] Henriquez G, Narayan M (2023). Targeting α-synuclein aggregation with immunotherapy: a promising therapeutic approach for Parkinson’s disease. Explor Neuroprot Ther, 3:207-234.

[111] Uemura N, Uemura MT, Luk KC, Lee VM-Y, Trojanowski JQ (2020). Cell-to-Cell Transmission of Tau and α-Synuclein. Trends Mol Med, 26:936-952. 10.1016/j.molmed.2020.03.012 PMC7529725 32371172

[112] Bergström A, Kallunki P, Fog K (2016). Development of Passive Immunotherapies for Synucleinopathies. Mov Disord, 31:203-213. 10.1002/mds.26481 26704735

[113] Schenk DB, Koller M, Ness DK, Griffith SG, Grundman M, Zago W, et al. (2017). First-in-human assessment of PRX002, an anti-α-synuclein monoclonal antibody, in healthy volunteers. Mov Disord, 32:211-218. 10.1002/mds.26878 PMC5324684 27886407

[114] Jankovic J, Goodman I, Safirstein B, Marmon TK, Schenk DB, Koller M, et al. (2018). Safety and Tolerability of Multiple Ascending Doses of PRX002/RG7935, an Anti-α-Synuclein Monoclonal Antibody, in Patients With Parkinson Disease. JAMA Neurol, 75:1206. 10.1001/jamaneurol.2018.1487 PMC6233845 29913017

[115] Pagano G, Boess FG, Taylor KI, Ricci B, Mollenhauer B, Poewe W, et al. (2021). A Phase II Study to Evaluate the Safety and Efficacy of Prasinezumab in Early Parkinson’s Disease (PASADENA): Rationale, Design, and Baseline Data. Front Neurol, 12:705407. 10.3389/fneur.2021.705407 PMC8518716 34659081

[116] Pagano G, Taylor KI, Anzures-Cabrera J, Marchesi M, Simuni T, Marek K, et al. (2022). Trial of Prasinezumab in Early-Stage Parkinson’s Disease. New England J Med, 387:421-432. 10.1056/NEJMoa2202867 35921451

[117] Weihofen A, Liu Y, Arndt JW, Huy C, Quan C, Smith BA, et al. (2019). Development of an aggregate-selective, human-derived α-synuclein antibody BIIB054 that ameliorates disease phenotypes in Parkinson’s disease models. Neurobiol Dis, 124:276-288. 10.1016/j.nbd.2018.10.016 30381260

[118] Brys M, Fanning L, Hung S, Ellenbogen A, Penner N, Yang M, et al. (2019). Randomized phase I clinical trial of anti-α-synuclein antibody BIIB054. Mov Disord, 34:1154-1163. 10.1002/mds.27738 PMC6771554 31211448

[119] Kuchimanchi M, Monine M, Kandadi Muralidharan K, Woodward C, Penner N (2020). Phase II Dose Selection for Alpha Synuclein-Targeting Antibody Cinpanemab (BIIB054) Based on Target Protein Binding Levels in the Brain. CPT Pharmacometrics Syst Pharmacol, 9:515-522. 10.1002/psp4.12538 PMC7499191 32613752

[120] Schofield DJ, Irving L, Calo L, Bogstedt A, Rees G, Nuccitelli A, et al. (2019). Preclinical development of a high affinity α-synuclein antibody, MEDI1341, that can enter the brain, sequester extracellular α-synuclein and attenuate α-synuclein spreading in vivo. Neurobiol Dis, 132:104582. 10.1016/j.nbd.2019.104582 31445162

[121] Nordström E, Eriksson F, Sigvardson J, Johannesson M, Kasrayan A, Jones-Kostalla M, et al. (2021). ABBV-0805, a novel antibody selective for soluble aggregated α-synuclein, prolongs lifespan and prevents buildup of α-synuclein pathology in mouse models of Parkinson’s disease. Neurobiol Dis, 161:105543. 10.1016/j.nbd.2021.105543 34737044

[122] Fjord-Larsen L, Thougaard A, Wegener KM, Christiansen J, Larsen F, Schrøder-Hansen LM, et al. (2021). Nonclinical safety evaluation, pharmacokinetics, and target engagement of Lu AF82422, a monoclonal IgG1 antibody against alpha-synuclein in development for treatment of synucleinopathies. MAbs, 13:1994690. 10.1080/19420862.2021.1994690 PMC8555527 34709986

[123] Cao L, Xiong S, Wu Z, Ding L, Zhou Y, Sun H, et al. (2021). Anti-Na + /K + -ATPase immunotherapy ameliorates α-synuclein pathology through activation of Na + /K + -ATPase α1-dependent autophagy. Sci Adv, 7: eabc5062. 10.1126/sciadv.abc5062 PMC7840131 33571110

[124] Huang S, Dong W, Lin X, Bian J (2024). Na+/K+-ATPase: ion pump, signal transducer, or cytoprotective protein, and novel biological functions. Neural Regen Res, 19:2684-2697. 10.4103/NRR.NRR-D-23-01175 PMC11168508 38595287

[125] Kumar AR, Kurup PA (2002). Inhibition of membrane Na+-K+ ATPase activity: a common pathway in central nervous system disorders. J Assoc Physicians India, 50:400-6. 11922232

[126] Cook JF, Hill DF, Snively BM, Boggs N, Suerken CK, Haq I, et al. (2014). Cognitive impairment in rapid-onset dystonia-parkinsonism. Mov Disord, 29:344-350. 10.1002/mds.25790 PMC3960305 24436111

[127] Felippe Gonçalves-de-Albuquerque C, Ribeiro Silva A, Ignácio da Silva C, Caire Castro-Faria-Neto H, Burth P (2017). Na/K Pump and Beyond: Na/K-ATPase as a Modulator of Apoptosis and Autophagy. Molecules, 22:578. 10.3390/molecules22040578 PMC6154632 28430151

[128] Zhu M, Cao L, Xiong S, Sun H, Wu Z, Bian J-S (2020). Na+/K+-ATPase-dependent autophagy protects brain against ischemic injury. Signal Transduct Target Ther, 5:55. 10.1038/s41392-020-0153-7 PMC7237650 32433549

[129] Allen SG, Meade RM, White Stenner LL, Mason JM (2023). Peptide-based approaches to directly target alpha-synuclein in Parkinson’s disease. Mol Neurodegener, 18:80. 10.1186/s13024-023-00675-8 PMC10633918 37940962

[130] Craik DJ, Fairlie DP, Liras S, Price D (2013). The Future of Peptide-based Drugs. Chem Biol Drug Des, 81:136-147. 10.1111/cbdd.12055 23253135

[131] Muttenthaler M, King GF, Adams DJ, Alewood PF (2021). Trends in peptide drug discovery. Nat Rev Drug Discov, 20:309-325. 10.1038/s41573-020-00135-8 33536635

[132] Meade RM, Allen SG, Williams C, Tang TMS, Crump MP, Mason JM (2023). An N-terminal alpha-synuclein fragment binds lipid vesicles to modulate lipid-induced aggregation. Cell Rep Phys Sci, 4:101563.

[133] Jin JW, Fan X, del Cid-Pellitero E, Liu X-X, Zhou L, Dai C, et al. (2021). Development of an α-synuclein knockdown peptide and evaluation of its efficacy in Parkinson’s disease models. Commun Biol, 4:232. 10.1038/s42003-021-01746-6 PMC7895943 33608634

[134] Horsley JR, Jovcevski B, Pukala TL, Abell AD (2022). Designer D-peptides targeting the N-terminal region of α-synuclein to prevent parkinsonian-associated fibrilization and cytotoxicity. Biochim Biophys Acta Proteins Proteom, 1870:140826. 10.1016/j.bbapap.2022.140826 35926717

[135] Sangwan S, Sahay S, Murray KA, Morgan S, Guenther EL, Jiang L, et al. (2020). Inhibition of synucleinopathic seeding by rationally designed inhibitors. Elife, 9:e46775. 10.7554/eLife.46775 PMC6977966 31895037

[136] Nim S, O’Hara DM, Corbi-Verge C, Perez-Riba A, Fujisawa K, Kapadia M, et al. (2023). Disrupting the α-synuclein-ESCRT interaction with a peptide inhibitor mitigates neurodegeneration in preclinical models of Parkinson’s disease. Nat Commun, 14:2150. 10.1038/s41467-023-37464-2 PMC10115881 37076542

[137] Popova B, Wang D, Rajavel A, Dhamotharan K, Lázaro DF, Gerke J, et al. (2021). Identification of Two Novel Peptides That Inhibit α-Synuclein Toxicity and Aggregation. Front Mol Neurosci, 14:659926. 10.3389/fnmol.2021.659926 PMC8072481 33912013

[138] Pirhaghi M, Frank SA, Alam P, Nielsen J, Sereikaite V, Gupta A, et al. (2022). A penetratin-derived peptide reduces the membrane permeabilization and cell toxicity of α-synuclein oligomers. J Biol Chem, 298:102688. 10.1016/j.jbc.2022.102688 PMC9791135 36370848

[139] Santos J, Gracia P, Navarro S, Peña-Díaz S, Pujols J, Cremades N, et al. (2021). α-Helical peptidic scaffolds to target α-synuclein toxic species with nanomolar affinity. Nat Commun, 12:3752. 10.1038/s41467-021-24039-2 PMC8213730 34145261

[140] Bernard K, Mota JA, Wene P, Corenblum MJ, Saez JL, Bartlett MJ, et al. (2024). The angiotensin (1-7) glycopeptide PNA5 improves cognition in a chronic progressive mouse model of Parkinson’s disease through modulation of neuroinflammation. Exp Neurol, 381:114926. 10.1016/j.expneurol.2024.114926 39153685

[141] Rabie MA, Abd El Fattah MA, Nassar NN, El-Abhar HS, Abdallah DM (2018). Angiotensin 1-7 ameliorates 6-hydroxydopamine lesions in hemiparkinsonian rats through activation of MAS receptor/PI3K/Akt/BDNF pathway and inhibition of angiotensin II type-1 receptor/NF-κB axis. Biochem Pharmacol, 151:126-134. 10.1016/j.bcp.2018.01.047 29428223

[142] Labandeira-Garcia JL, Labandeira CM, Guerra MJ, Rodriguez-Perez AI (2024). The role of the brain renin-angiotensin system in Parkinson′s disease. Transl Neurodegener, 13:22. 10.1186/s40035-024-00410-3 PMC11017622 38622720

[143] Gao Q, Chen R, Wu L, Huang Q, Wang X-X, Tian Y-Y, et al. (2022). Angiotensin-(1-7) reduces α-synuclein aggregation by enhancing autophagic activity in Parkinson’s disease. Neural Regen Res, 17:1138. 10.4103/1673-5374.324854 PMC8552854 34558543

[144] McGlinchey RP, Lee JC (2015). Cysteine cathepsins are essential in lysosomal degradation of α-synuclein. Proc Natl Acad Sci USA, 112:9322-9327. 10.1073/pnas.1500937112 PMC4522768 26170293

[145] Matsuki A, Watanabe Y, Hashimoto S, Hoshino A, Matoba S (2024). Cathepsin L prevents the accumulation of alpha-synuclein fibrils in the cell. Genes to Cells, 29:328-336. 10.1111/gtc.13099 38366711

[146] Tong Y, Zhu W, Chen J, Zhang W, Xu F, Pang J (2023). Targeted Degradation of Alpha-Synuclein by Autophagosome-Anchoring Chimera Peptides. J Med Chem, 66:12614-12628. 10.1021/acs.jmedchem.3c01303 37652467

[147] Kilpeläinen TP, Hellinen L, Vrijdag J, Yan X, Svarcbahs R, Vellonen K-S, et al. (2020). The effect of prolyl oligopeptidase inhibitors on alpha-synuclein aggregation and autophagy cannot be predicted by their inhibitory efficacy. Biomed Pharmacother, 128:110253. 10.1016/j.biopha.2020.110253 32447211

[148] Svarcbahs R, Julku UH, Norrbacka S, Myöhänen TT (2018). Removal of prolyl oligopeptidase reduces alpha-synuclein toxicity in cells and in vivo. Sci Rep, 8:1552. 10.1038/s41598-018-19823-y PMC5784134 29367610

[149] Myöhänen T, Hannula M, Van Elzen R, Gerard M, Van Der Veken P, García-Horsman J, et al. (2012). A prolyl oligopeptidase inhibitor, KYP-2047, reduces α-synuclein protein levels and aggregates in cellular and animal models of Parkinson’s disease. Br J Pharmacol, 166:1097-1113. 10.1111/j.1476-5381.2012.01846.x PMC3417432 22233220

[150] Savolainen MH, Richie CT, Harvey BK, Männistö PT, Maguire-Zeiss KA, Myöhänen TT (2014). The beneficial effect of a prolyl oligopeptidase inhibitor, KYP-2047, on alpha-synuclein clearance and autophagy in A30P transgenic mouse. Neurobiol Dis, 68:1-15. 10.1016/j.nbd.2014.04.003 PMC7254878 24746855

[151] Savolainen MH, Yan X, Myöhänen TT, Huttunen HJ (2015). Prolyl Oligopeptidase Enhances α-Synuclein Dimerization via Direct Protein-Protein Interaction. J Biol Chem, 290:5117-5126. 10.1074/jbc.M114.592931 PMC4335246 25555914

[152] Rostami J, Jäntti M, Cui H, Rinne MK, Kukkonen JP, Falk A, et al. (2020). Prolyl oligopeptidase inhibition by KYP-2407 increases alpha-synuclein fibril degradation in neuron-like cells. Biomed Pharmacother, 131:110788. 10.1016/j.biopha.2020.110788 33152946

[153] Wang Z-Y, Liu J, Zhu Z, Su C-F, Sreenivasmurthy SG, Iyaswamy A, et al. (2021). Traditional Chinese medicine compounds regulate autophagy for treating neurodegenerative disease: A mechanism review. Biomed Pharmacother, 133:110968. 10.1016/j.biopha.2020.110968 33189067

[154] Xia M-L, Xie X-H, Ding J-H, Du R-H, Hu G (2020). Astragaloside IV inhibits astrocyte senescence: implication in Parkinson’s disease. J Neuroinflamm, 17:105. 10.1186/s12974-020-01791-8 PMC7137443 32252767

[155] Bhattacharjee A, Abuammar H, Juhász G (2024). Lysosomal activity depends on TRPML1-mediated Ca2+ release coupled to incoming vesicle fusions. J Biol Chem, 300:107911. 10.1016/j.jbc.2024.107911 PMC11599452 39433126

[156] Zhang X, Chen W, Gao Q, Yang J, Yan X, Zhao H, et al. (2019). Rapamycin directly activates lysosomal mucolipin TRP channels independent of mTOR. PLoS Biol, 17:e3000252. 10.1371/journal.pbio.3000252 PMC6528971 31112550

[157] Wu L-K, Agarwal S, Kuo C-H, Kung Y-L, Day CH, Lin P-Y, et al. (2022). Artemisia Leaf Extract protects against neuron toxicity by TRPML1 activation and promoting autophagy/mitophagy clearance in both in vitro and in vivo models of MPP+/MPTP-induced Parkinson’s disease. Phytomedicine, 104:154250. 10.1016/j.phymed.2022.154250 35752074

[158] Baskin J, Jeon JE, Lewis SJG (2021). Nanoparticles for drug delivery in Parkinson’s disease. J Neurol, 268:1981-1994. 10.1007/s00415-020-10291-x 33141248

[159] Gao G, Chen R, He M, Li J, Li J, Wang L, et al. (2019). Gold nanoclusters for Parkinson’s disease treatment. Biomaterials, 194:36-46. 10.1016/j.biomaterials.2018.12.013 30576972

[160] Srivastava AK, Roy Choudhury S, Karmakar S (2020). Melatonin/polydopamine nanostructures for collective neuroprotection-based Parkinson’s disease therapy. Biomater Sci, 8:1345-1363. 10.1039/c9bm01602c 31912833

[161] Grancharova T, Simeonova S, Pilicheva B, Zagorchev P (2024). Gold Nanoparticles in Parkinson’s Disease Therapy: A Focus on Plant-Based Green Synthesis. Cureus, 16:e54671. 10.7759/cureus.54671 PMC10960252 38524031

[162] Ghazy E, Rahdar A, Barani M, Kyzas GZ (2021). Nanomaterials for Parkinson disease: Recent progress. J Mol Struct, 1231:129698.

[163] Liu H, Han Y, Wang T, Zhang H, Xu Q, Yuan J, et al. (2020). Targeting Microglia for Therapy of Parkinson’s Disease by Using Biomimetic Ultrasmall Nanoparticles. J Am Chem Soc, 142:21730-21742. 10.1021/jacs.0c09390 33315369

[164] Liu H, Zheng Q, Yuan J, Gao Y, Wang T, Zhang H, et al. (2023). Modulating SQSTM1/p62-dependent selective autophagy of neurons by activating Nrf2 with multifunctional nanoparticles to eliminate α-synuclein aggregates and boost therapy of Parkinson’s disease. Nano Today, 49:101770.

[165] Tsai JM, Nowak RP, Ebert BL, Fischer ES (2024). Targeted protein degradation: from mechanisms to clinic. Nat Rev Mol Cell Biol, 25:740-757. 10.1038/s41580-024-00729-9 38684868

[166] Zhao L, Zhao J, Zhong K, Tong A, Jia D (2022). Targeted protein degradation: mechanisms, strategies and application. Signal Transduct Target Ther, 7:113. 10.1038/s41392-022-00966-4 PMC8977435 35379777

[167] Ding Y, Xing D, Fei Y, Luo S, Lu B (2024). Perspectives of autophagy-tethering compounds (ATTECs) in drug discovery. Medicine Plus, 1:100004.

[168] Bao J, Chen Z, Li Y, Chen L, Wang W, Sheng C, et al. (2024). Discovery of Novel PDEδ Autophagic Degraders: A Case Study of Autophagy-Tethering Compound (ATTEC). ACS Med Chem Lett, 15:29-35. 10.1021/acsmedchemlett.3c00161 PMC10788939 38229750

[169] Keefe AD, Pai S, Ellington A (2010). Aptamers as therapeutics. Nat Rev Drug Discov, 9:537-550. 10.1038/nrd3141 PMC7097324 20592747

[170] Liao X, Qin G, Liu Z, Ren J, Qu X (2024). Bioorthogonal Aptamer-ATTEC Conjugates for Degradation of Alpha-Synuclein via Autophagy-Lysosomal Pathway. Small, 20:e2306760. 10.1002/smll.202306760 37821404

[171] Ji CH, Kim HY, Lee MJ, Heo AJ, Park DY, Lim S, et al. (2022). The AUTOTAC chemical biology platform for targeted protein degradation via the autophagy-lysosome system. Nat Commun, 13:904. 10.1038/s41467-022-28520-4 PMC8850458 35173167

[172] Levin J, Sing N, Melbourne S, Morgan A, Mariner C, Spillantini MG, et al. (2022). Safety, tolerability and pharmacokinetics of the oligomer modulator anle138b with exposure levels sufficient for therapeutic efficacy in a murine Parkinson model: A randomised, double-blind, placebo-controlled phase 1a trial. EBioMedicine, 80:104021. 10.1016/j.ebiom.2022.104021 PMC9065877 35500536

[173] Antonschmidt L, Matthes D, Dervişoğlu R, Frieg B, Dienemann C, Leonov A, et al. (2022). The clinical drug candidate anle138b binds in a cavity of lipidic α-synuclein fibrils. Nat Commun, 13:5385. 10.1038/s41467-022-32797-w PMC9474542 36104315

[174] Lee J, Sung KW, Bae E-J, Yoon D, Kim D, Lee JS, et al. (2023). Targeted degradation of α-synuclein aggregates in Parkinson’s disease using the AUTOTAC technology. Mol Neurodegener, 18:41. 10.1186/s13024-023-00630-7 PMC10290391 37355598

[175] Watanabe Y, Taguchi K, Tanaka M (2023). Roles of Stress Response in Autophagy Processes and Aging-Related Diseases. Int J Mol Sci, 24:13804. 10.3390/ijms241813804 PMC10531041 37762105

[176] Cuervo AM, Stefanis L, Fredenburg R, Lansbury PT, Sulzer D (2004). Impaired Degradation of Mutant α-Synuclein by Chaperone-Mediated Autophagy. Science (1979), 305:1292-1295. 10.1126/science.1101738 15333840

[177] Stykel MG, Humphries KM, Kamski-Hennekam E, Buchner-Duby B, Porte-Trachsel N, Ryan T, et al. (2021). α-Synuclein mutation impairs processing of endomembrane compartments and promotes exocytosis and seeding of α-synuclein pathology. Cell Rep, 35:109099. 10.1016/j.celrep.2021.109099 33979611

[178] Nascimento AC, Erustes AG, Reckziegel P, Bincoletto C, Ureshino RP, Pereira GJS, et al. (2020). α-Synuclein Overexpression Induces Lysosomal Dysfunction and Autophagy Impairment in Human Neuroblastoma SH-SY5Y. Neurochem Res, 45:2749-2761. 10.1007/s11064-020-03126-8 32915398

[179] Chen J-Y, Zhu Q, Cai C-Z, Luo H-B, Lu J-H (2022). α-mangostin derivative 4e as a PDE4 inhibitor promote proteasomal degradation of alpha-synuclein in Parkinson’s disease models through PKA activation. Phytomedicine, 101:154125. 10.1016/j.phymed.2022.154125 35525236

[180] Liu Y, Levine B (2015). Autosis and autophagic cell death: the dark side of autophagy. Cell Death Differ, 22:367-376. 10.1038/cdd.2014.143 PMC4326571 25257169

[181] Kroemer G, Levine B (2008). Autophagic cell death: the story of a misnomer. Nat Rev Mol Cell Biol, 9:1004-1010. 10.1038/nrm2527 PMC2727358 18971948

[182] Qiu S, Wang J, Huang S, Sun S, Zhang Z, Bao N (2018). Overactive autophagy is a pathological mechanism underlying premature suture ossification in nonsyndromic craniosynostosis. Sci Rep, 8:6525. 10.1038/s41598-018-24885-z PMC5916928 29695736

[183] Zhang K, Wang F, Zhai M, He M, Hu Y, Feng L, et al. (2023). Hyperactive neuronal autophagy depletes BDNF and impairs adult hippocampal neurogenesis in a corticosterone-induced mouse model of depression. Theranostics, 13:1059-1075. 10.7150/thno.81067 PMC9925310 36793868

